# Supplementary figures and images for: Potential effect of tolvaptan on polycystic liver disease for patients with ADPKD meeting the Japanese criteria of tolvaptan use
Source: PLoS One. 2022 Feb 17;17(2):e0264065. doi: 10.1371/journal.pone.0264065 (PMC8853523; doi:10.1371/journal.pone.0264065)

**Premenopausal female**

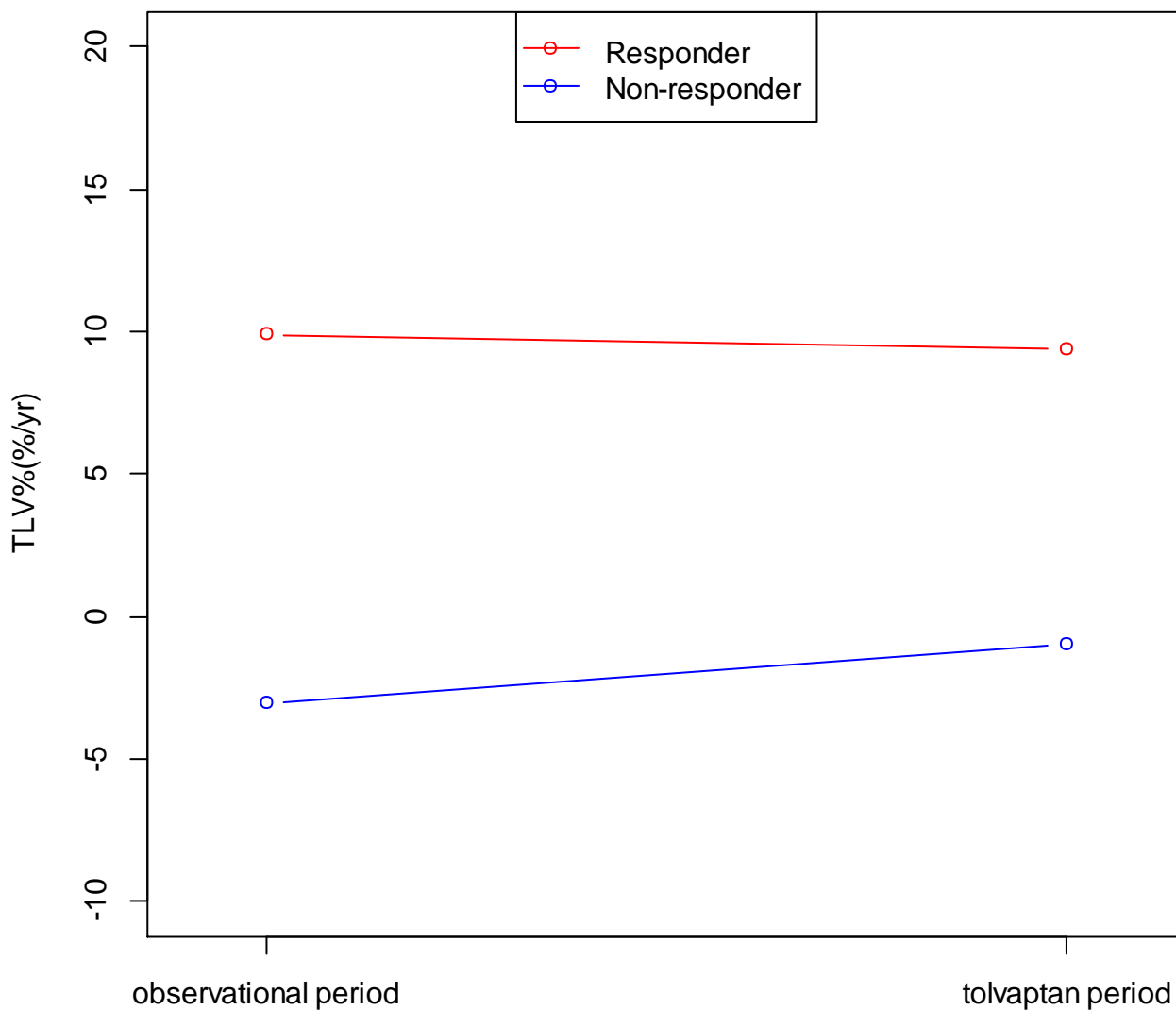

**Postmenopausal female**

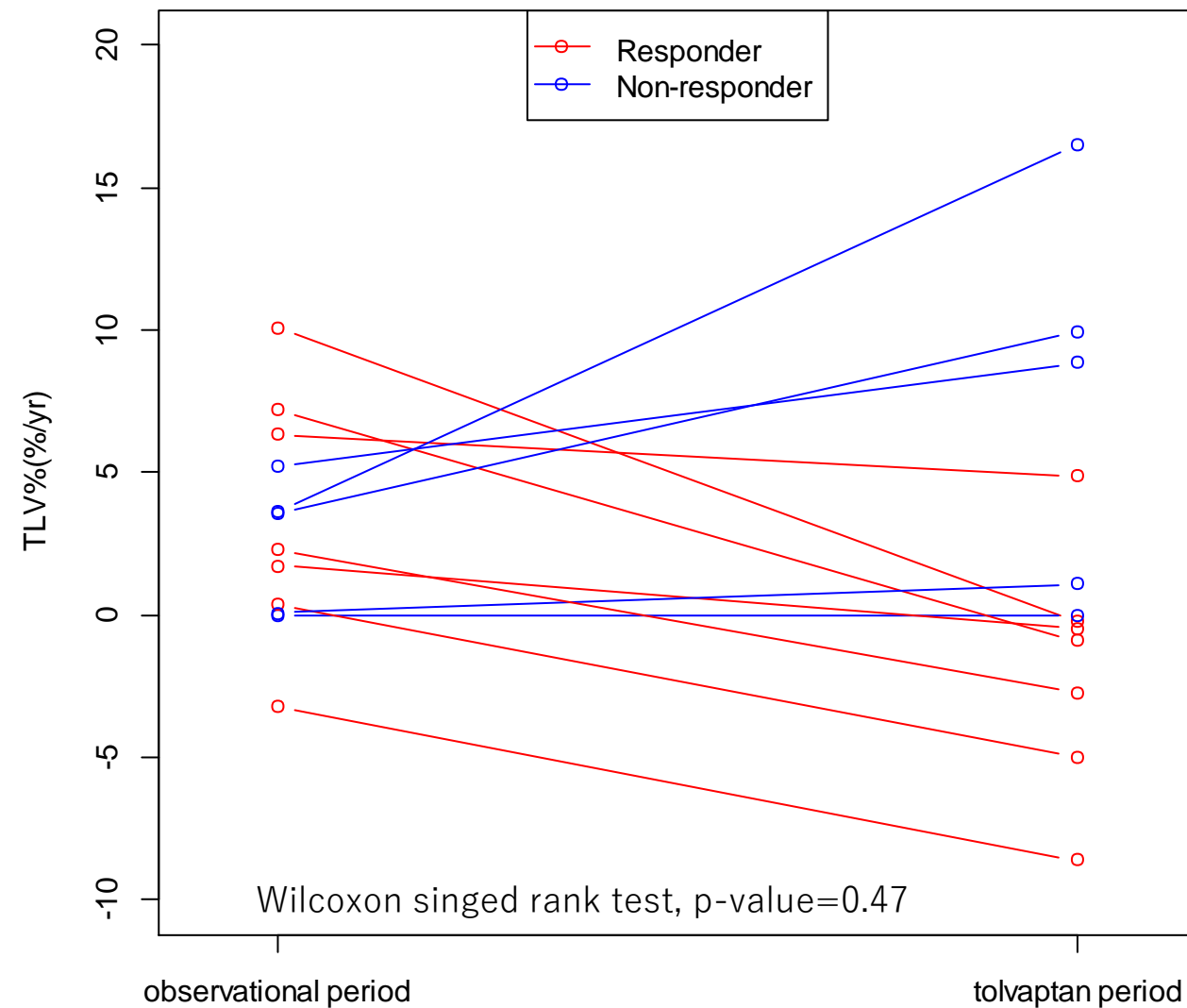

Supplement: S2 Fig — The annual growth rate of total liver volume was not change in premenopausal female(n = 2) and in postmenopausal female (n = 12). Red circle represents the responder defined as the patients whose annual liver growth rate of total liver volume decreased after tolvaptan use, while the blue circle represents the non-responder defined as the patients whose annual liver growth rate of total liver volume increased. Abbreviation. TLV%: annual growth rate of total liver volume. (PDF) [file pone.0264065.s002.pdf]

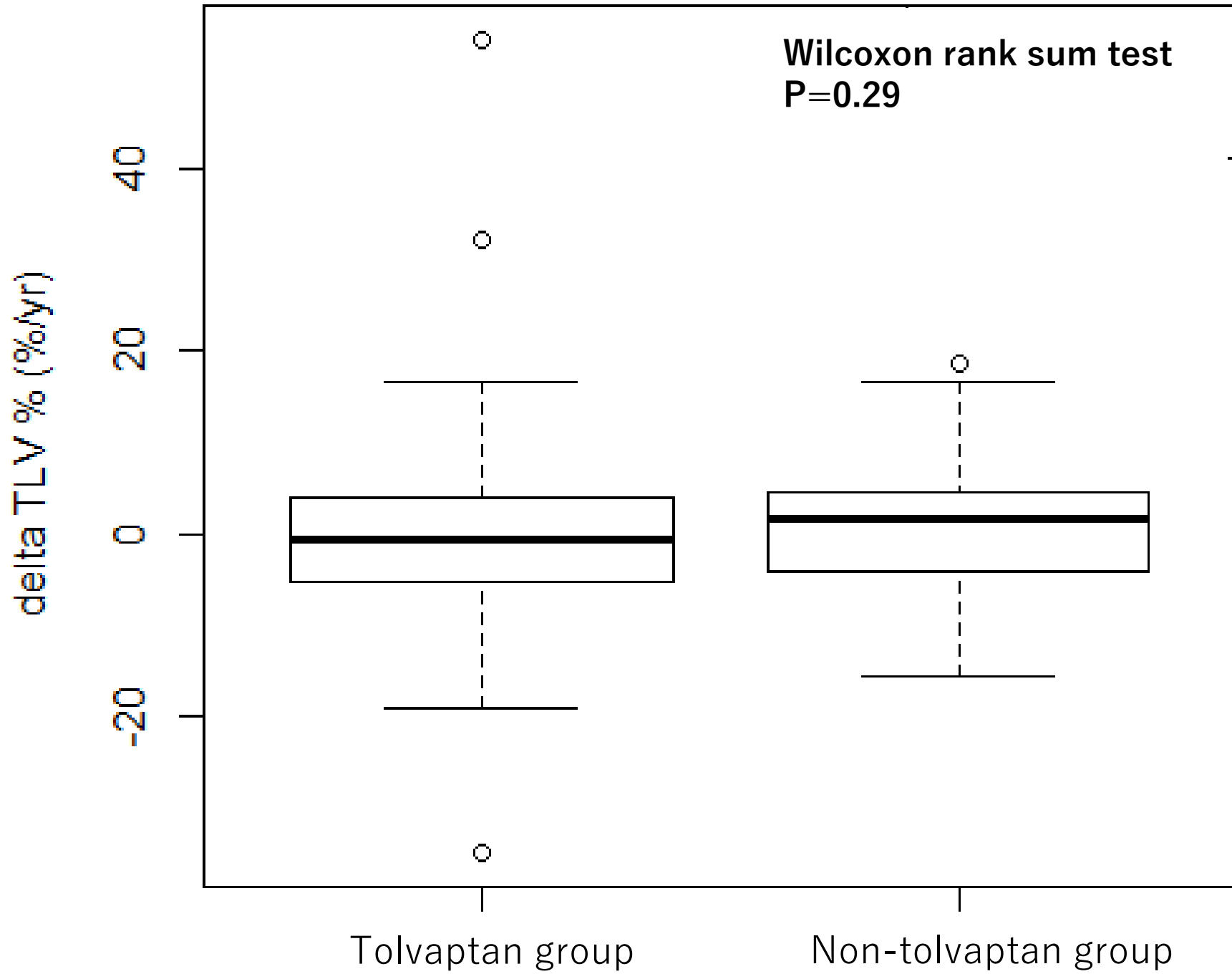

Supplement: S3 Fig — The median change in the annual growth rate of total liver volume (TLV) was not statistically different between the tolvaptan group (-0.7 (range -35.0, 54.2) %/year) and the non-tolvaptan group (1.7 (-15.6, 18.7) %/year) (p = 0.29, Wilcoxon rank sum test). ΔTLV %: change in annual growth rate of TLV. (PDF) [file pone.0264065.s003.pdf]

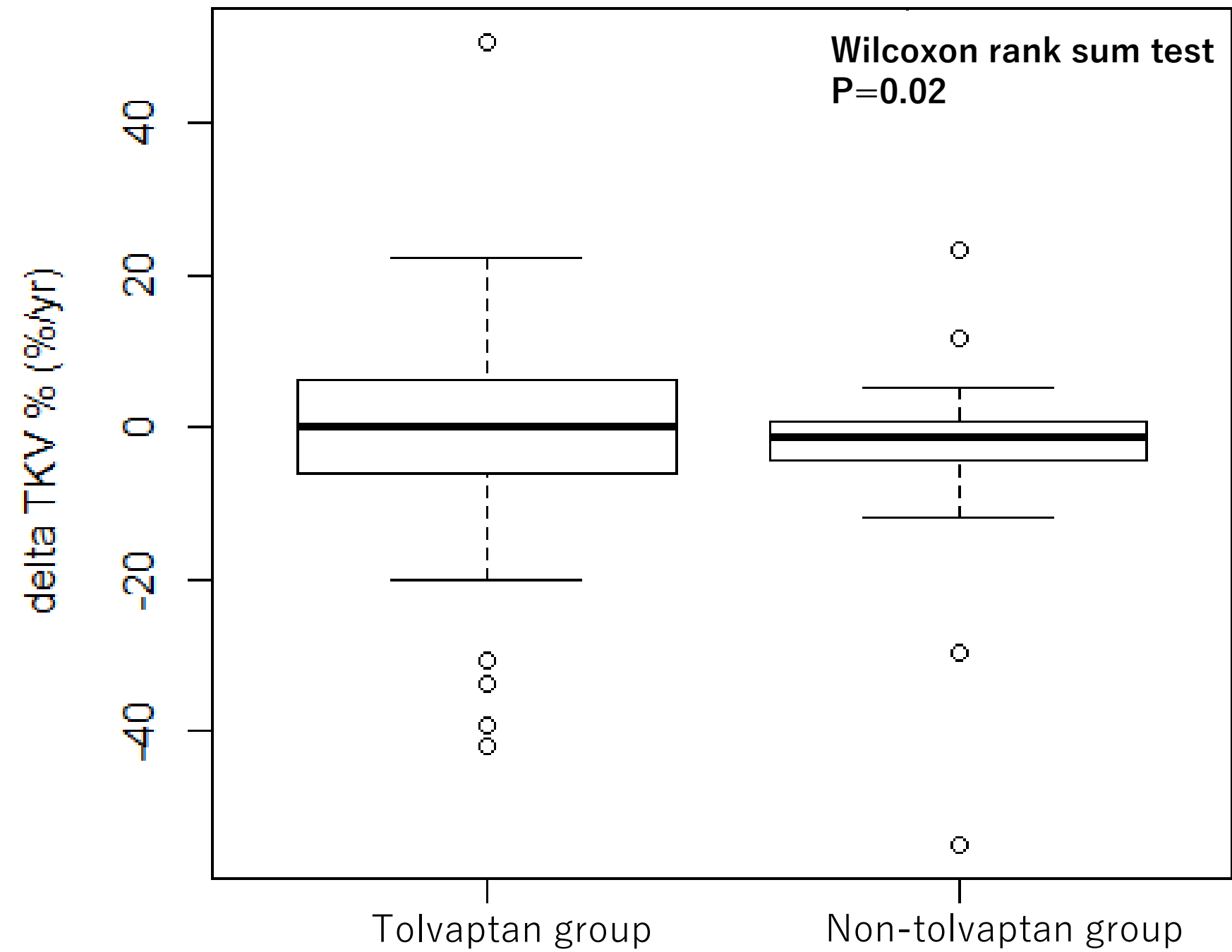

Supplement: S4 Fig — The median difference in the annual growth rate in total kidney volume (TKV) was larger in the tolvaptan group (0.2 (-42.0, 50.9)) than in the non-tolvaptan group (1.1 (-55.0, 23.4)) (p = 0.02, Wilcoxon rank sum test). Abbreviation. ΔTKV%: the change in growth rate of TKV. (PDF) [file pone.0264065.s004.pdf]
