# Supplementary material for: Potential effect of tolvaptan on polycystic liver disease for patients with ADPKD meeting the Japanese criteria of tolvaptan use
Source: PLoS One. 2022 Feb 17;17(2):e0264065. doi: 10.1371/journal.pone.0264065 (PMC8853523; doi:10.1371/journal.pone.0264065)
Supplement: S1 Table — (DOCX) [file pone.0264065.s005.docx]

**S1 Table. The baseline demographic and laboratory data of the tolvaptan group and non-tolvaptan group in patients without the history of interventions for polycystic liver**

|  | | | Tolvaptan group | | Non-tolvaptan group | |  |  |
| --- | --- | --- | --- | --- | --- | --- | --- | --- |
|  | | | n=82 | | n=25 | | p value | |
| Baseline characteristics | | |  |  |  |  |  |  |
|  | Male | n(%) | 28 | (34.1) | 11 | (44.0) | 0.37 |  |
|  | Age | (years old) | 54.4 | ±10.4 | 50.3 | ±13.4 | 0.22 |  |
|  | Height | (cm) | 163.8 | ±8.8 | 165.1 | ±9.9 | 0.54 |  |
|  | Body weight | (kg) | 61.8 | ±2.4 | 64.6 | ±11.3 | 0.30 |  |
|  | Body-mass index | (kg/m2) | 23.0 | ±0.6 | 23.7 | ±4.0 | 0.40 |  |
|  | Systolic blood pressure | (mmHg) | 126.4 | ±3.2 | 128.0 | ±15.4 | 0.53 |  |
|  | Diastolic blood pressure | (mmHg) | 79.3 | ±2.2 | 81.7 | ±12.0 | 0.31 |  |
|  | Height adjusted total liver volume | (mL/m) | 1746 | (557-7432) | 1359 | (640-12507) | 0.81 |  |
|  | Height adjusted total kidney volume | (mL/m) | 905 | (227-4152) | 633 | (405-1978) | <0.01 | ** |
| Comorbidities, n(%) | | |  |  |  |  |  |  |
|  | Hypertension | n(%) | 67 | (81.7) | 19 | (76.0) | 0.59 |  |
|  | Diabetes mellitus | n(%) | 2 | (2.4) | 0 | (0.0) | 0.42 |  |
| Intervention, n(%) | | | 36 | (43.9) | 9 | (36.0) | 0.53 |  |
|  | Cyst drainage for infection | n(%) | 3 | (3.7) | 0 | (0.0) | 0.32 |  |
|  | Cyst drainage for mass reduction | n(%) | 16 | (19.5) | 6 | (24.0) | 0.49 |  |
|  | Drainage volume | (mL) | 656 | (140-2575) | 1100 | (450-3385) | <0.01 | ** |
|  | Trans-arterial embolization for liver | n(%) | 25 | (30.5) | 7 | (28.0) | 0.82 |  |
| Medications, n(%) | | |  |  |  |  |  |  |
|  | Angiotensin converting enzyme inhibitor or angiotensin II receptor blocker | n(%) | 56 | (68.3) | 17 | (68.0) | 0.97 |  |
|  | Ursodeoxycholic acid | n(%) | 15 | (18.3) | 5 | (20.0) | 0.91 |  |
| Laboratory values (serum) | | |  |  |  |  |  |  |
|  | Platelet count | (*10^3^/μL) | 209.6 | ±13.1 | 238.0 | ±68.4 | 0.10 |  |
|  | Albumin | (g/dL) | 3.7 | ±0.1 | 4.1 | ±0.4 | **<0.01** | ****** |
|  | Aspartate aminotrasferase | (IU/L) | 19.4 | ±1.6 | 20.5 | ±5.3 | 0.38 |  |
|  | Alanine aminotransferase | (IU/L) | 15.5 | ±1.8 | 20.5 | ±19.9 | 0.23 |  |
|  | Alkaline phosphatase | (IU/L) | 232.4 | ±23.1 | 222.7 | ±111.3 | 0.80 |  |
|  | Gamma glutamyltransferase | (IU/L) | 48.6 | ±9.5 | 56.6 | ±48.8 | 0.44 |  |
|  | Total bilirubin | (mg/dL) | 0.7 | ±0.1 | 0.7 | ±0.2 | 0.84 |  |
|  | Uric acid | (mg/dL) | 6.1 | ±0.3 | 5.6 | ±1.6 | 0.24 |  |
|  | Creatinine | (mg/dL) | 1.1 | ±0.1 | 0.9 | ±0.3 | **<0.01** | ****** |
|  | eGFR | (mL/min/1.73m^2^) | 51.6 | ±20.2 | 65.9 | ±24.1 | **0.02** | ***** |
|  | Prothrombin time | (%) | 95.3 | ±2.9 | 88.0 | ±27.6 | 0.33 |  |
| Laboratory values (urine) | | |  |  |  |  |  |  |
|  | Hematuria | n(%) | 22 | (26.8) | 5 | (20.0) | 0.57 |  |
|  | Proteinuria | (g/gCre) | 0.08 | (0.05-0.16) | 0.08 | (0.01-1.03) | 0.59 |  |
|  | N-acetyl-D-glucosamine | (U/mL) | 5.7 | ±0.9 | 6.0 | ±3.6 | 0.81 |  |
