# Supplementary material for: Potential effect of tolvaptan on polycystic liver disease for patients with ADPKD meeting the Japanese criteria of tolvaptan use
Source: PLoS One. 2022 Feb 17;17(2):e0264065. doi: 10.1371/journal.pone.0264065 (PMC8853523; doi:10.1371/journal.pone.0264065)
Supplement: S2 Table — (DOCX) [file pone.0264065.s006.docx]

**S2 Table. The baseline demographic and laboratory data of responders and non-responders in patients without the history of interventions for polycystic live**

|  |  |  | Responder | | Non-responder | |  |  |
| --- | --- | --- | --- | --- | --- | --- | --- | --- |
|  |  |  | n=44 | | n=38 | | p value | |
| Baseline characteristics | | |  |  |  |  |  |  |
|  | Male | n(%) | 13 | (29.5) | 15 | (39.5) | 0.34 |  |
|  | Age | (years old) | 57.9 | ±9.8 | 50.3 | ±9.5 | **<0.01** | ****** |
|  | Height | (cm) | 161.8 | ±7.7 | 165.8 | ±9.8 | **0.05** | ***** |
|  | Body weight | (kg) | 60.9 | ±9.3 | 62.9 | ±11.5 | 0.39 |  |
|  | Body-mass index | (kg/m2) | 23.2 | ±2.7 | 22.7 | ±2.7 | 0.45 |  |
|  | Systolic blood pressure | (mmHg) | 126.2 | ±13.4 | 126.7 | ±16.9 | 0.88 |  |
|  | Diastolic blood pressure | (mmHg) | 77.3 | ±10.4 | 81.7 | ±10.3 | 0.06 |  |
|  | Height adjusted total liver volume | (mL/m) | 1068 | (557-6691) | 918 | (640-4177) | 0.05 |  |
|  | Height adjusted total kidney volume | (mL/m) | 994 | (450-4152) | 513 | (405-1928) | 0.34 |  |
|  | Annual growth rate of TLV | (%/year) | 5.9 | (-4.7-46.6) | 3.4 | (-12.8-26.7) | <0.01 |  |
|  | Annual growth rate of TKV | (%/year) | 4.5 | (-3.5-27.9) | -0.9 | (-55.2-15.2) | 0.12 |  |
|  | post menopausal female | n(%) | 18 | (85.7) | 11 | (84.6) | 0.93 |  |
| Dose of tolvaptan | | (mg/day) | 66.6 | ±33.3 | 71.1 | ±27.5 | 0.52 |  |
| Comorbidities, n(%) | | |  |  |  |  |  |  |
|  | Hypertension | n(%) | 39 | (88.6) | 28 | (73.7) | 0.08 |  |
|  | Diabetes mellitus | n(%) | 1 | (2.3) | 1 | (2.6) | 0.94 |  |
| Intervention | | | 24 | (54.5) | 12 | (31.6) | **0.04** |  |
|  | Cyst drainage for infection | n(%) | 3 | (6.8) | 0 | (0.0) | 0.10 |  |
|  | Cyst drainage for mass reduction | n(%) | 11 | (25.0) | 5 | (13.2) | 0.18 |  |
|  | Drainage volume | (mL) | 656 | (139-2575) | 595 | (250-1066) | **<0.01** | ****** |
|  | Trans-arterial embolization for liver | n(%) | 16 | (36.4) | 9 | (23.7) | 0.21 |  |
| Medications | | |  |  |  |  |  |  |
|  | Angiotensin converting enzyme inhibitor or angiotensin II receptor blocker | n(%) | 34 | (77.3) | 22 | (57.9) | 0.06 |  |
|  | Ursodeoxycholic acid | n(%) | 13 | (29.5) | 2 | (5.3) | **<0.01** | ****** |
| Laboratory values (serum) | | |  |  |  |  |  |  |
|  | Platelet count | (*103/μL) | 200.4 | ±69.6 | 220.2 | ±45.8 | 0.13 |  |
|  | Albumin | (g/dL) | 3.7 | ±0.4 | 3.8 | ±0.4 | 0.41 |  |
|  | Aspartate aminotrasferase | (IU/L) | 19.8 | ±8.5 | 19.0 | ±6.0 | 0.62 |  |
|  | Alanine aminotransferase | (IU/L) | 14.5 | ±7.3 | 16.8 | ±9.0 | 0.22 |  |
|  | Alkaline phosphatase | (IU/L) | 259.8 | ±120.4 | 200.6 | ±76.6 | **<0.01** | ****** |
|  | Gamma glutamyltransferase | (IU/L) | 50.1 | ±41.8 | 46.9 | ±46.1 | 0.75 |  |
|  | Total bilirubin | (mg/dL) | 0.7 | ±0.4 | 0.8 | ±0.3 | 0.73 |  |
|  | Uric acid | (mg/dL) | 6.1 | ±1.5 | 6.0 | ±1.3 | 0.62 |  |
|  | Creatinine | (mg/dL) | 1.1 | ±0.5 | 1.2 | ±0.4 | 0.77 |  |
|  | eGFR | (mL/min/1.73m2) | 51.8 | ±21.3 | 51.5 | ±18.8 | 0.95 |  |
|  | Prothrombin time | (%) | 94.0 | ±13.5 | 96.8 | ±13.0 | 0.35 |  |
| Laboratory values (urine) | | |  |  |  |  |  |  |
|  | Hematuria | n(%) | 8 | (18.2) | 5 | (13.2) | 0.53 |  |
|  | Proteinuria | (g/gCre) | 0.08 | (0.01-1.69) | 0.06 | (0.01-0.25) | 0.60 |  |
|  | N-acetyl-D-glucosamine | (U/mL) | 5.9 | ±2.9 | 5.5 | ±5.3 | 0.72 |  |
