# Supplementary material for: Potential effect of tolvaptan on polycystic liver disease for patients with ADPKD meeting the Japanese criteria of tolvaptan use
Source: PLoS One. 2022 Feb 17;17(2):e0264065. doi: 10.1371/journal.pone.0264065 (PMC8853523; doi:10.1371/journal.pone.0264065)
Supplement: S3 Table — (DOCX) [file pone.0264065.s007.docx]

**S3 Table. Logistic regression model analyzing the prognostic factors of the growth rate of TLV in ADPKD in patients without the history of interventions for polycystic liver**

|  |  | Unadjusted | | | Adjusted | | |
| --- | --- | --- | --- | --- | --- | --- | --- |
|  |  | Odds | 95%CI | p-value | Odds | 95%CI | p-value |
| Male |  | 0.94 | (0.33-2.70) | 0.91 | 1.47 | (0.06-36.78) | 0.81 |
| Age | (10year) | 4.01 | (1.68-9.56) | <0.01 | 1.33 | (0.23-7.61) | 0.75 |
| Body-mass index |  | 1.07 | (0.88-1.30) | 0.49 | 1.10 | (0.68-1.75) | 0.70 |
| Height adjusted total kidney volume | (100mL/m) | 0.93 | (0.87-0.99) | 0.83 | 0.87 | (0.66-1.14) | 0.31 |
| Annual change of total liver volume | (%/year) | 1.23 | (1.08-1.40) | <0.01 | 1.17 | (1.01-1.36) | 0.04 |
| Ursodeoxychcolic acid |  | 10.37 | (1.24-86.58) | 0.03 | 10.83 | (0.66-176.76) | 0.09 |
| Drainage volume | (100mL) | 1.07 | (0.90-1.27) | 0.45 | 1.10 | (0.85-1.43) | 0.46 |

Abbreviations. ADPKD: autosomal dominant polycystic kidney disease. TLV: total liver volume.
