# Supplementary material for: Potential effect of tolvaptan on polycystic liver disease for patients with ADPKD meeting the Japanese criteria of tolvaptan use
Source: PLoS One. 2022 Feb 17;17(2):e0264065. doi: 10.1371/journal.pone.0264065 (PMC8853523; doi:10.1371/journal.pone.0264065)
Supplement: S1 Dataset — (DOCX) [file pone.0264065.s008.docx]

| Case | Group | Dose of tolvaptan | Baseline characteristics | | | | | | | | | | | Comorbidities | | | Medications | | | Physical intervention for PLD | | | | Menstrual status | |
| --- | --- | --- | --- | --- | --- | --- | --- | --- | --- | --- | --- | --- | --- | --- | --- | --- | --- | --- | --- | --- | --- | --- | --- | --- | --- |
|  |  |  | Sex | Age | Height | BW | BMI | SBP | DBP | MBP | htTLV | htTKV | Hypertension | | Diabetes mellitus | ACEi or ARB | | UDCA | TAE | | Drainage for infection | Drainage of non- infection |  | |  |
|  |  | (mg/day) |  | (years old) | (cm) | (kg) |  | (mmHg) | (mmHg) | (mmHg) | (mL/m) | (mL/m) |  |  |  |  |  |  |  |  |  |  |  |  |  |
| 1 | Tolvaptan | 15 | female | 45 | 155 | 51.2 | 21.4 | 118 | 79 | 92.0 | 1083 | 1331 | Yes | | No | Yes | | No | No | | No | No | NA | |  |
| 2 | Tolvaptan | 15 | female | 52 | 147 | 47.5 | 22.1 | 152 | 79 | 103.3 | 2271 | 1616 | Yes | | No | Yes | | No | No | | No | No | NA | |  |
| 3 | Tolvaptan | 15 | male | 58 | 175 | 75.0 | 24.5 | 148 | 100 | 116.0 | 1745 | 4152 | Yes | | No | Yes | | No | No | | No | No | NA | |  |
| 4 | Tolvaptan | 15 | male | 68 | 170 | 64.4 | 22.2 | 120 | 72 | 88.0 | 749 | 647 | Yes | | No | Yes | | No | No | | No | No | NA | |  |
| 5 | Tolvaptan | 15 | female | 73 | 155 | 54.9 | 22.9 | 126 | 84 | 98.0 | 3735 | 1756 | Yes | | No | Yes | | No | No | | No | No | after menopause | |  |
| 6 | Tolvaptan | 15 | female | 76 | 158 | 67.1 | 26.8 | 122 | 66 | 84.7 | 1590 | 3515 | Yes | | Yes | No | | No | No | | No | No | after menopause | |  |
| 7 | Tolvaptan | 15 | female | 77 | 150 | 65.4 | 29.2 | 126 | 64 | 84.7 | 629 | 1376 | Yes | | No | Yes | | No | No | | No | No | after menopause | |  |
| 8 | Tolvaptan | 45 | male | 55 | 173 | 61.0 | 20.4 | 126 | 83 | 97.3 | 676 | 3395 | Yes | | No | Yes | | Yes | No | | No | No | NA | |  |
| 9 | Tolvaptan | 45 | female | 58 | 158 | 54.2 | 21.8 | 168 | 91 | 116.7 | 2422 | 477 | No | | No | Yes | | No | No | | No | No | after menopause | |  |
| 10 | Tolvaptan | 60 | male | 33 | 167 | 64.0 | 22.8 | 144 | 101 | 115.3 | 940 | 902 | No | | No | No | | No | No | | No | No | NA | |  |
| 11 | Tolvaptan | 60 | male | 36 | 173 | 69.8 | 23.3 | 124 | 85 | 98.0 | 970 | 1134 | No | | No | No | | No | No | | No | No | NA | |  |
| 12 | Tolvaptan | 60 | male | 38 | 180 | 67.2 | 20.7 | 171 | 91 | 117.7 | 658 | 808 | No | | No | No | | No | No | | No | No | NA | |  |
| 13 | Tolvaptan | 60 | male | 39 | 170 | 67.2 | 23.4 | 132 | 80 | 97.3 | 910 | 545 | Yes | | No | Yes | | No | No | | No | No | NA | |  |
| 14 | Tolvaptan | 60 | female | 40 | 154 | 52.9 | 22.3 | 129 | 89 | 102.3 | 903 | 681 | No | | No | No | | No | No | | No | No | before menopause | |  |
| 15 | Tolvaptan | 60 | male | 41 | 172 | 87.0 | 29.4 | 115 | 74 | 87.7 | 1599 | 892 | Yes | | No | No | | No | No | | No | No | NA | |  |
| 16 | Tolvaptan | 60 | male | 42 | 179 | 81.7 | 25.5 | 109 | 71 | 83.7 | 1084 | 1083 | Yes | | No | Yes | | No | No | | No | No | NA | |  |
| 17 | Tolvaptan | 60 | male | 43 | 174 | 76.7 | 25.3 | 110 | 73 | 85.3 | 1308 | 587 | Yes | | No | Yes | | No | No | | No | No | NA | |  |
| 18 | Tolvaptan | 60 | male | 43 | 168 | 79.2 | 28.1 | 134 | 84 | 100.7 | 1273 | 910 | Yes | | Yes | Yes | | No | No | | No | No | NA | |  |
| 19 | Tolvaptan | 60 | female | 44 | 161 | 67.8 | 26.2 | 125 | 90 | 101.7 | 744 | 2076 | Yes | | No | Yes | | No | No | | No | No | NA | |  |
| 20 | Tolvaptan | 60 | male | 45 | 182 | 68.2 | 20.5 | 125 | 82 | 96.3 | 734 | 1112 | Yes | | No | Yes | | No | No | | No | No | NA | |  |
| 21 | Tolvaptan | 60 | female | 48 | 160 | 48.0 | 18.7 | 116 | 65 | 82.0 | 944 | 1241 | No | | No | No | | No | No | | No | No | NA | |  |
| 22 | Tolvaptan | 60 | female | 49 | 154 | 41.4 | 17.4 | 112 | 73 | 86.0 | 1782 | 570 | No | | No | No | | No | No | | No | No | NA | |  |
| 23 | Tolvaptan | 60 | female | 49 | 163 | 58.9 | 22.3 | 122 | 76 | 91.3 | 1307 | 672 | Yes | | No | No | | No | No | | No | No | NA | |  |
| 24 | Tolvaptan | 60 | male | 50 | 167 | 70.2 | 25.1 | 98 | 65 | 76.0 | 861 | 450 | Yes | | No | Yes | | No | No | | No | No | NA | |  |
| 25 | Tolvaptan | 60 | male | 50 | 165 | 64.9 | 23.8 | 95 | 68 | 77.0 | 718 | 834 | Yes | | No | No | | No | No | | No | No | NA | |  |
| 26 | Tolvaptan | 60 | female | 51 | 151 | 47.0 | 20.6 | 125 | 78 | 93.7 | 905 | 738 | Yes | | No | Yes | | No | No | | No | No | before menopause | |  |
| 27 | Tolvaptan | 60 | female | 51 | 151 | 50.4 | 22.3 | 122 | 78 | 92.7 | 1066 | 932 | Yes | | No | No | | No | No | | No | No | NA | |  |
| 28 | Tolvaptan | 60 | female | 53 | 164 | 61.2 | 22.8 | 117 | 58 | 77.7 | 756 | 700 | Yes | | No | Yes | | No | No | | No | No | after menopause | |  |
| 29 | Tolvaptan | 60 | male | 57 | 170 | 71.9 | 24.9 | 129 | 86 | 100.3 | 718 | 909 | No | | No | No | | No | No | | No | No | NA | |  |
| 30 | Tolvaptan | 60 | female | 66 | 160 | 52.3 | 20.4 | 152 | 103 | 119.3 | 1709 | 1338 | Yes | | No | No | | No | No | | No | No | after menopause | |  |
| 31 | Tolvaptan | 60 | female | 69 | 162 | 53.5 | 20.4 | 127 | 79 | 95.0 | 3097 | 1559 | Yes | | No | Yes | | No | No | | No | No | after menopause | |  |
| 32 | Tolvaptan | 90 | male | 43 | 184 | 87.7 | 25.8 | 135 | 92 | 106.3 | 826 | 2536 | Yes | | No | Yes | | No | No | | No | No | NA | |  |
| 33 | Tolvaptan | 90 | male | 44 | 181 | 66.9 | 20.4 | 113 | 74 | 87.0 | 890 | 1803 | Yes | | No | Yes | | No | No | | No | No | NA | |  |
| 34 | Tolvaptan | 90 | female | 47 | 163 | 54.2 | 20.4 | 133 | 84 | 100.3 | 676 | 562 | Yes | | No | Yes | | No | No | | No | No | NA | |  |
| 35 | Tolvaptan | 90 | male | 48 | 175 | 73.2 | 23.8 | 112 | 79 | 90.0 | 879 | 490 | Yes | | No | Yes | | No | No | | No | No | NA | |  |
| 36 | Tolvaptan | 90 | male | 49 | 178 | 60.7 | 19.2 | 114 | 68 | 83.3 | 557 | 507 | Yes | | No | Yes | | No | No | | No | No | NA | |  |
| 37 | Tolvaptan | 90 | male | 50 | 175 | 85.3 | 28.0 | 132 | 78 | 96.0 | 1115 | 1187 | No | | No | No | | No | No | | No | No | NA | |  |
| 38 | Tolvaptan | 90 | female | 54 | 155 | 53.5 | 22.4 | 123 | 93 | 103.0 | 1291 | 879 | Yes | | No | Yes | | No | No | | No | No | after menopause | |  |
| 39 | Tolvaptan | 90 | male | 56 | 173 | 84.0 | 28.1 | 134 | 92 | 106.0 | 6691 | 2021 | Yes | | No | Yes | | No | No | | No | No | NA | |  |
| 40 | Tolvaptan | 90 | female | 59 | 160 | 41.8 | 16.3 | 124 | 83 | 96.7 | 679 | 518 | Yes | | No | Yes | | No | No | | No | No | after menopause | |  |
| 41 | Tolvaptan | 120 | male | 46 | 185 | 79.5 | 23.2 | 136 | 84 | 101.3 | 1103 | 1354 | Yes | | No | Yes | | No | No | | No | No | NA | |  |
| 42 | Tolvaptan | 120 | female | 47 | 165 | 55.2 | 20.4 | 139 | 91 | 107.0 | 1071 | 1750 | Yes | | No | Yes | | No | No | | No | No | NA | |  |
| 43 | Tolvaptan | 120 | female | 52 | 164 | 54.0 | 20.1 | 133 | 94 | 107.0 | 3380 | 1057 | No | | No | No | | No | No | | No | No | after menopause | |  |
| 44 | Tolvaptan | 120 | female | 57 | 166 | 55.7 | 20.3 | 136 | 89 | 104.7 | 1269 | 899 | Yes | | No | Yes | | No | No | | No | No | after menopause | |  |
| 45 | Tolvaptan | 120 | male | 63 | 170 | 62.0 | 21.5 | 136 | 81 | 99.3 | 1363 | 2190 | Yes | | No | No | | No | No | | No | No | NA | |  |
| 46 | Tolvaptan | 120 | female | 67 | 164 | 66.7 | 25.0 | 112 | 67 | 82.0 | 1629 | 1339 | Yes | | No | Yes | | No | No | | No | No | after menopause | |  |
| 47 | Non-tolvaptan |  | male | 33 | 169 | 67.2 | 23.5 | 119 | 107 | 111.0 | 915 | 481 | Yes | | No | Yes | | No | No | | No | No | NA | |  |
| 48 | Non-tolvaptan |  | male | 34 | 170 | 70.0 | 24.2 | 124 | 69 | 87.3 | 980 | 522 | Yes | | No | Yes | | No | No | | No | No | NA | |  |
| 49 | Non-tolvaptan |  | female | 35 | 155 | 56.0 | 23.3 | 110 | 79 | 89.3 | 2434 | 616 | Yes | | No | No | | No | No | | No | No | before menopause | |  |
| 50 | Non-tolvaptan |  | male | 35 | 178 | 63.3 | 19.9 | 113 | 72 | 85.7 | 665 | 463 | No | | No | No | | No | No | | No | No | NA | |  |
| 51 | Non-tolvaptan |  | female | 36 | 165 | 48.0 | 17.6 | 120 | 90 | 100.0 | 747 | 633 | No | | No | No | | No | No | | No | No | before menopause | |  |
| 52 | Non-tolvaptan |  | male | 39 | 176 | 60.0 | 19.4 | 126 | 76 | 92.7 | 921 | 635 | Yes | | No | Yes | | No | No | | No | No | NA | |  |
| 53 | Non-tolvaptan |  | male | 41 | 190 | 84.0 | 23.3 | 130 | 85 | 100.0 | 938 | 482 | Yes | | No | Yes | | No | No | | No | No | NA | |  |
| 54 | Non-tolvaptan |  | male | 41 | 172 | 65.8 | 22.2 | 121 | 76 | 91.0 | 754 | 405 | Yes | | No | Yes | | No | No | | No | No | NA | |  |
| 55 | Non-tolvaptan |  | female | 42 | 156 | 51.0 | 21.0 | NA | NA | NA | 4177 | 495 | No | | No | No | | No | No | | No | No | before menopause | |  |
| 56 | Non-tolvaptan |  | male | 45 | 183 | 61.0 | 18.2 | 135 | 87 | 103.0 | 1824 | 471 | Yes | | No | Yes | | No | No | | No | No | NA | |  |
| 57 | Non-tolvaptan |  | male | 56 | 166 | 66.0 | 24.0 | 93 | 60 | 71.0 | 730 | 504 | Yes | | No | Yes | | No | No | | No | No | NA | |  |
| 58 | Non-tolvaptan |  | female | 64 | 154 | 65.5 | 27.6 | 112 | 73 | 86.0 | 1168 | 505 | Yes | | No | Yes | | No | No | | No | No | after menopause | |  |
| 59 | Non-tolvaptan |  | female | 65 | 142 | 49.2 | 24.3 | 130 | 66 | 87.3 | 705 | 1134 | Yes | | No | Yes | | No | No | | No | No | after menopause | |  |
| 60 | Non-tolvaptan |  | male | 66 | 166 | 76.0 | 27.6 | 146 | 88 | 107.3 | 701 | 1928 | Yes | | No | No | | No | No | | No | No | NA | |  |
| 61 | Non-tolvaptan |  | female | 73 | 153 | 46.5 | 19.9 | 120 | 70 | 86.7 | 1359 | 810 | No | | No | No | | No | No | | No | No | after menopause | |  |
| 62 | Non-tolvaptan |  | male | 75 | 163 | 66.0 | 24.8 | 128 | 68 | 88.0 | 640 | 1004 | Yes | | No | Yes | | No | No | | No | No | NA | |  |
| 63 | Tolvaptan | 15 | female | 64 | 157 | 55.9 | 22.8 | 119 | 72 | 87.7 | 2087 | 960 | Yes | | No | Yes | | No | No | | No | Yes | Yes | |  |
| 64 | Tolvaptan | 15 | female | 73 | 152 | 47.8 | 20.7 | 157 | 95 | 115.7 | 5422 | 1125 | Yes | | No | Yes | | Yes | Yes | | No | No | Yes | |  |
| 65 | Tolvaptan | 30 | female | 51 | 158 | 55.9 | 22.3 | 135 | 81 | 99 | 5283 | 227 | Yes | | No | Yes | | Yes | Yes | | No | No | NA | |  |
| 66 | Tolvaptan | 30 | female | 57 | 159 | 59.4 | 23.6 | 119 | 81 | 93.7 | 4359 | 1277 | Yes | | No | Yes | | No | Yes | | No | No | Yes | |  |
| 67 | Tolvaptan | 30 | female | 71 | 158 | 66.4 | 26.6 | 122 | 74 | 90 | 3482 | 773 | Yes | | No | No | | Yes | Yes | | Yes | No | Yes | |  |
| 68 | Tolvaptan | 30 | male | 81 | 171 | 75.0 | 25.6 | 116 | 89 | 98 | 799 | 933 | Yes | | No | No | | No | No | | No | Yes | NA | |  |
| 69 | Tolvaptan | 45 | female | 48 | 164 | 65.9 | 24.7 | 121 | 72 | 88.3 | 4171 | 828 | Yes | | NA | Yes | | No | Yes | | No | No | NA | |  |
| 70 | Tolvaptan | 45 | female | 65 | 159 | 55.6 | 22.0 | 120 | 69 | 86 | 4769 | 1614 | Yes | | No | No | | No | Yes | | Yes | No | Yes | |  |
| 71 | Tolvaptan | 60 | female | 40 | 160 | 66.8 | 26.3 | 124 | 54 | 77.3 | 6352 | 473 | Yes | | No | No | | Yes | Yes | | No | No | No | |  |
| 72 | Tolvaptan | 60 | female | 47 | 160 | 57.0 | 22.3 | 112 | 70 | 84 | 2511 | 623 | No | | No | No | | No | Yes | | No | No | NA | |  |
| 73 | Tolvaptan | 60 | male | 47 | 176 | 72.6 | 23.5 | 135 | 85 | 101.7 | 6780 | 463 | No | | No | No | | No | Yes | | No | No | NA | |  |
| 74 | Tolvaptan | 60 | female | 48 | 159 | 62.5 | 24.8 | 101 | 76 | 84.3 | 2266 | 1037 | Yes | | No | Yes | | No | Yes | | No | Yes | NA | |  |
| 75 | Tolvaptan | 60 | male | 48 | 169 | 69.0 | 24.2 | 116 | 74 | 88 | 5798 | 586 | Yes | | No | Yes | | Yes | No | | No | Yes | NA | |  |
| 76 | Tolvaptan | 60 | female | 52 | 168 | 63.0 | 22.5 | 117 | 83 | 94.3 | 711 | 2398 | Yes | | No | Yes | | No | No | | No | Yes | NA | |  |
| 77 | Tolvaptan | 60 | female | 53 | 155 | 54.8 | 22.8 | 116 | 80 | 92 | 2289 | 535 | Yes | | No | Yes | | Yes | Yes | | No | No | NA | |  |
| 78 | Tolvaptan | 60 | female | 55 | 152 | 60.6 | 26.2 | 138 | 100 | 112.7 | 2398 | 686 | Yes | | No | Yes | | No | No | | No | Yes | NA | |  |
| 79 | Tolvaptan | 60 | female | 57 | 162 | 49.9 | 19.0 | 93 | 63 | 73 | 2674 | 412 | Yes | | No | Yes | | No | No | | No | Yes | Yes | |  |
| 80 | Tolvaptan | 60 | female | 58 | 153 | 51.8 | 22.2 | 156 | 89 | 111.3 | 4550 | 656 | No | | No | No | | No | Yes | | No | No | Yes | |  |
| 81 | Tolvaptan | 60 | female | 72 | 155 | 55.0 | 22.8 | 118 | 63 | 81.3 | 2523 | 479 | Yes | | No | No | | Yes | Yes | | No | No | Yes | |  |
| 82 | Tolvaptan | 90 | female | 49 | 164 | 59.6 | 22.2 | 114 | 71 | 85.3 | 4599 | 771 | Yes | | No | Yes | | No | Yes | | No | No | NA | |  |
| 83 | Tolvaptan | 90 | female | 49 | 165 | 74.5 | 27.5 | 135 | 87 | 103 | 5120 | 1263 | Yes | | No | No | | No | No | | No | Yes | No | |  |
| 84 | Tolvaptan | 90 | female | 52 | 169 | 58.0 | 20.2 | 133 | 94 | 107 | 5116 | 1242 | Yes | | No | Yes | | No | Yes | | No | No | NA | |  |
| 85 | Tolvaptan | 90 | female | 54 | 168 | 55.3 | 19.5 | 101 | 69 | 79.7 | 3157 | 461 | Yes | | No | Yes | | No | Yes | | No | No | NA | |  |
| 86 | Tolvaptan | 90 | male | 55 | 164 | 69.9 | 25.9 | 127 | 79 | 95 | 7432 | 1152 | No | | No | Yes | | No | Yes | | No | No | NA | |  |
| 87 | Tolvaptan | 90 | female | 57 | 146 | 49.6 | 23.3 | 141 | 87 | 105 | 2084 | 806 | Yes | | No | No | | No | No | | No | Yes | Yes | |  |
| 88 | Tolvaptan | 90 | female | 59 | 163 | 58.8 | 22.1 | 128 | 84 | 98.7 | 1748 | 546 | Yes | | No | Yes | | No | Yes | | No | Yes | Yes | |  |
| 89 | Tolvaptan | 90 | female | 64 | 154 | 53.6 | 22.7 | 123 | 64 | 83.7 | 3461 | 981 | Yes | | No | Yes | | Yes | Yes | | No | Yes | Yes | |  |
| 90 | Tolvaptan | 90 | female | 65 | 165 | 56.3 | 20.7 | 111 | 70 | 83.7 | 5716 | 626 | Yes | | No | Yes | | Yes | Yes | | No | No | Yes | |  |
| 91 | Tolvaptan | 90 | female | 66 | 158 | 52.0 | 21.0 | 136 | 80 | 98.7 | 1848 | 1629 | Yes | | No | Yes | | Yes | Yes | | No | Yes | Yes | |  |
| 92 | Tolvaptan | 90 | female | 66 | 159 | 64.0 | 25.3 | 131 | 75 | 93.7 | 4953 | 683 | No | | No | Yes | | Yes | No | | Yes | No | Yes | |  |
| 93 | Tolvaptan | 120 | female | 44 | 171 | 65.9 | 22.5 | 122 | 75 | 90.7 | 3405 | 590 | Yes | | No | Yes | | No | No | | No | Yes | No | |  |
| 94 | Tolvaptan | 120 | female | 49 | 164 | 69.7 | 26.1 | 132 | 85 | 100.7 | 4143 | 1171 | Yes | | No | Yes | | No | No | | No | Yes | NA | |  |
| 95 | Tolvaptan | 120 | female | 58 | 157 | 43.6 | 17.6 | 109 | 79 | 89 | 4353 | 1024 | Yes | | No | Yes | | Yes | Yes | | No | No | Yes | |  |
| 96 | Tolvaptan | 120 | male | 62 | 152 | 60.5 | 26.3 | 161 | 67 | 98.3 | 3166 | 487 | Yes | | No | Yes | | Yes | Yes | | No | No | NA | |  |
| 97 | Tolvaptan | 120 | female | 69 | 150 | 55.2 | 24.6 | 127 | 59 | 81.7 | 2773 | 768 | Yes | | NA | Yes | | Yes | Yes | | No | Yes | Yes | |  |
| 98 | Tolvaptan | 120 | female | 71 | 163 | 56.8 | 21.4 | 149 | 93 | 111.7 | 5098 | 1244 | Yes | | No | Yes | | No | Yes | | No | Yes | Yes | |  |
| 99 | Non-tolvaptan |  | female | 41 | 159 | 85.9 | 34.0 | 130 | 85 | 100 | 12507 | 832 | Yes | | No | Yes | | No | Yes | | No | No | NA | |  |
| 100 | Non-tolvaptan |  | female | 42 | 165 | 88.4 | 32.5 | 165 | 104 | 124.3 | 8983 | 514 | Yes | | No | Yes | | Yes | Yes | | No | Yes | NA | |  |
| 101 | Non-tolvaptan |  | female | 47 | 166 | 49.5 | 17.9 | NA | NA | NA | 4344 | 485 | No | | No | No | | No | Yes | | No | Yes | NA | |  |
| 102 | Non-tolvaptan |  | female | 47 | 167 | 75.5 | 27.1 | 129 | 84 | 99 | 8646 | 1019 | Yes | | No | Yes | | Yes | Yes | | No | No | NA | |  |
| 103 | Non-tolvaptan |  | female | 49 | 169 | 70.7 | 24.7 | 149 | 101 | 117 | 3092 | 878 | Yes | | No | Yes | | Yes | Yes | | No | Yes | NA | |  |
| 104 | Non-tolvaptan |  | female | 50 | 158 | 67.7 | 27.0 | 139 | 88 | 105 | 8597 | 1123 | Yes | | No | Yes | | Yes | Yes | | No | No | NA | |  |
| 105 | Non-tolvaptan |  | male | 66 | 165 | 65.5 | 24.2 | 120 | 80 | 93.3 | 3503 | 831 | No | | No | No | | No | No | | No | Yes | NA | |  |
| 106 | Non-tolvaptan |  | Female | 66 | 164 | 56.7 | 21.1 | 130 | 78 | 95.3 | 5552 | 749 | Yes | | No | Yes | | No | Yes | | No | Yes | NA | |  |
| 107 | Non-tolvaptan |  | Female | 71 | 157 | 59.4 | 24.0 | 156 | 93 | 114 | 2136 | 1978 | Yes | | No | Yes | | Yes | No | | No | Yes | NA | |  |

Abbreviation: BW; body weight, BMI; body mass index, SBP; systolic blood pressure, DBP; diastolic blood pressure, MBP; mean blood pressure, htTLV; height adjusted total liver volume, htTKV; height adjusted total kidney volume, ACEi; angiotensin-converting enzyme inhibitors, ARB; angiotensin II receptor blockers, UDCA; ursodeoxycholic acid, TAE; trans-arterial embolization for liver artery, PLD; polycystic liver disease, NA; not available.

| Case | Laboratory values(serum) | | | | | | | | | | | Laboratory data(urine) | | | Change in annual growth rate | | Crude annual growth rate | | | | | Responder | | | |
| --- | --- | --- | --- | --- | --- | --- | --- | --- | --- | --- | --- | --- | --- | --- | --- | --- | --- | --- | --- | --- | --- | --- | --- | --- | --- |
|  | Plt | Alb | AST | ALT | ALP | γGTP | bil | UA | Cre | eGFR | PT | Hematuria | Proteinuira | NAG | Liver | Kidney | Liver | | Kidney | | | Liver | | Kidney | |
|  |  |  |  |  |  |  |  |  |  |  |  |  |  |  |  |  | observational period | tolvaptan period | observational period | tolvaptan period |  | |  | |  |
|  | (*10^3^/μL) | (g/dL) | (IU/L) | (IU/L) | (IU/L) | (IU/L) | (mg  /dL) | (mg  /dL) | (mg/dL) | (mL/min/1.73m^2^) | (%) |  | (g/gCre) | (U  /mL) | (%  /year) | (%  /year) | (%/year) | (%/year) | (%/year) | (%/year) |  | |  | |  |
| 1 | 233 | 3.4 | 44 | 13 | 124 | 16 | 0.7 | 4.3 | 1.46 | 32.6 | 103.8 | 0 | 0.13 | 6.2 | 3.9 | 7.6 | -0.1 | 3.8 | -1.9 | 5.8 | No | | No | |  |
| 2 | 206 | 3.7 | 18 | 14 | 180 | 37 | 0.4 | 8.1 | 2.14 | 20.5 | 90 | 0 | 0.32 | 4.8 | -11.1 | -20.0 | 14.3 | 3.2 | 26.3 | 6.3 | Yes | | Yes | |  |
| 3 | 129 | 3.7 | 18 | 6 | 190 | 27 | 1 | 8.4 | 2.48 | 23 | 91.5 | 0 | 0.17 | 6.8 | -0.1 | NA | 2.9 | 2.8 | NA | 8.8 | Yes | | NA | |  |
| 4 | 203 | 4.3 | 19 | 13 | 161 | 14 | 0.3 | 6.1 | 1.88 | 29.3 | 134.9 | 0 | 0.06 | 9 | -4.8 | 5.0 | -0.7 | -5.4 | 9.4 | 14.4 | Yes | | No | |  |
| 5 | 206 | 4.1 | 27 | 14 | 297 | 108 | 1 | 7.1 | 2.36 | 16.6 | 109.8 | 0 | 1.69 | 10.4 | -1.5 | 8.0 | 6.3 | 4.9 | -2.6 | 5.3 | Yes | | No | |  |
| 6 | 189 | 3.7 | 21 | 13 | 364 | 253 | 0.6 | 4 | 1.67 | 16.7 | 99.5 | 0 | 0.55 | 12 | 0.1 | 9.3 | 0.0 | 0.0 | 12.8 | 22.1 | No | | No | |  |
| 7 | 161 | 3.4 | 17 | 8 | 154 | 20 | 0.6 | 7 | 1.71 | 23.2 | 94 | 0 | 0.06 | 6.3 | -10.3 | -7.5 | 10.1 | -0.2 | 8.7 | 1.2 | Yes | | Yes | |  |
| 8 | 174 | 3.9 | 15 | 12 | 245 | 15 | 0.6 | 7.6 | 1.74 | 25.1 | 103.8 | 1 | 0.39 | 9.6 | -2.6 | 1.9 | 0.9 | -1.7 | -1.0 | 1.0 | Yes | | No | |  |
| 9 | 177 | 4.1 | 18 | 12 | 210 | 60 | 0.6 | 6 | 1.56 | 27.6 | 112 | 1 | 0.29 | 6.3 | -2.2 | -10.5 | 1.7 | -0.5 | 3.7 | -6.7 | Yes | | Yes | |  |
| 10 | 322 | 3.8 | 21 | 27 | 145 | 33 | 0.7 | 6.8 | 0.79 | 95.5 | 107.4 | 0 | 0.05 | 4 | 4.4 | -30.7 | -0.9 | 3.4 | 9.2 | -21.5 | No | | Yes | |  |
| 11 | 219 | 4.1 | 16 | 13 | 154 | 27 | 0.8 | 6.3 | 1.03 | 67.7 | 92 | 0 | 0.05 | 2.3 | -1.9 | -9.4 | -1.6 | -3.6 | 10.3 | 1.0 | Yes | | Yes | |  |
| 12 | 188 | 4.5 | 18 | 21 | 61 | 18 | 1.7 | 5.4 | 1.08 | 63.8 | 104.1 | 0 | 0.07 | 5.2 | 1.7 | -0.1 | 2.0 | 3.7 | 3.1 | 3.0 | No | | Yes | |  |
| 13 | 308 | 4.4 | 16 | 9 | 122 | 16 | 0.6 | 5.3 | 0.86 | 81.8 | 113.8 | 0 | 0.09 | 2.1 | 3.5 | -2.4 | -1.6 | 2.0 | 6.1 | 3.8 | No | | Yes | |  |
| 14 | 220 | 4.2 | 14 | 9 | 177 | 12 | 0.5 | 5 | 0.91 | 56 | 87.5 | 0 | 0.12 | 3 | 2.1 | -8.5 | -3.0 | -0.9 | -0.6 | -9.1 | No | | Yes | |  |
| 15 | 202 | 4.7 | 16 | 15 | 152 | 58 | 0.4 | 7 | 0.78 | 88.3 | 95.3 | 0 | 0.08 | NA | 6.8 | 2.3 | -0.9 | 5.9 | 4.3 | 6.6 | No | | No | |  |
| 16 | 273 | 3.9 | 28 | 51 | 140 | 54 | 0.8 | 6.8 | 0.97 | 70.6 | 102.9 | 0 | 0.02 | 3.2 | 0.2 | 17.8 | 2.0 | 2.1 | -4.3 | 13.6 | No | | No | |  |
| 17 | 275 | 3.8 | 18 | 21 | 148 | 43 | 0.9 | 5.7 | 0.9 | 76.6 | 101.2 | 0 | 0.03 | 4.5 | 16.8 | 2.0 | 0.8 | 17.6 | 3.7 | 5.7 | No | | No | |  |
| 18 | 230 | 3.9 | 12 | 14 | 114 | 29 | 0.7 | 6.8 | 0.93 | 73.9 | 104.8 | 0 | 0.03 | 8.3 | -15.8 | -1.2 | 11.2 | -4.6 | 9.5 | 8.3 | Yes | | Yes | |  |
| 19 | 253 | 3.5 | 17 | 10 | 173 | 22 | 0.6 | 8.7 | 1.58 | 29.9 | 119.3 | 1 | 0.17 | 3.9 | 2.4 | 2.3 | -2.0 | 0.4 | 5.1 | 7.4 | No | | No | |  |
| 20 | 238 | 3.9 | 18 | 12 | 214 | 23 | 0.7 | 5.9 | 1.21 | 54.2 | 93 | 0 | 0.06 | 4.4 | 3.8 | 7.1 | -0.5 | 3.2 | 0.8 | 7.9 | No | | No | |  |
| 21 | 272 | 3.3 | 14 | 9 | 200 | 10 | 0.7 | 4.9 | 0.92 | 52.7 | 107.2 | 0 | 0.08 | 4.3 | 15.4 | 0.0 | -6.9 | 8.5 | 2.0 | 2.0 | No | | No | |  |
| 22 | 224 | 3.8 | 24 | 19 | 123 | 38 | 0.8 | 4.8 | 0.91 | 54 | 108.5 | 1 | 0.11 | 0 | 4.2 | 4.1 | 3.2 | 7.4 | -1.1 | 3.0 | No | | No | |  |
| 23 | 272 | 4.3 | 19 | 17 | 229 | 35 | 0.7 | 3.8 | 0.7 | 69.7 | 88.9 | 0 | 0.13 | 3 | -5.3 | 2.1 | 8.9 | 3.5 | 0.9 | 3.0 | Yes | | No | |  |
| 24 | 273 | 4.3 | 19 | 26 | 258 | 37 | 0.5 | 6.6 | 1.09 | 58.5 | NA | 0 | 0.06 | NA | 2.2 | -11.0 | -2.7 | -0.5 | 6.1 | -4.9 | No | | Yes | |  |
| 25 | 173 | 3.9 | 26 | 30 | 135 | 18 | 0.6 | 6.7 | 1.03 | 62.6 | 100.3 | 0 | 0.07 | 2.4 | 7.8 | 0.2 | -2.4 | 5.4 | 1.0 | 1.2 | No | | No | |  |
| 26 | 227 | 4.2 | 21 | 16 | 121 | 16 | 0.8 | 4.2 | 0.57 | 86.8 | 96.6 | 0 | 0.06 | 4.8 | -0.5 | -2.3 | 9.9 | 9.4 | -4.7 | -7.0 | Yes | | Yes | |  |
| 27 | 324 | 4.1 | 17 | 13 | 211 | 18 | 0.5 | 3 | 0.68 | 71.5 | 116.2 | 0 | 0.13 | 7.7 | -12.0 | -16.0 | 16.3 | 4.3 | 11.0 | -5.0 | Yes | | Yes | |  |
| 28 | 244 | 3.7 | 14 | 7 | 157 | 9 | 0.7 | 4.6 | 0.73 | 66.2 | 107.4 | 0 | 0.02 | 0 | -5.4 | -5.6 | -3.2 | -8.6 | 7.5 | 2.0 | Yes | | Yes | |  |
| 29 | 228 | 3.6 | 16 | 13 | 187 | 25 | 0.4 | 5.4 | 0.95 | 65.7 | 109.3 | 1 | 0.02 | 1.7 | -3.1 | 0.6 | 4.4 | 1.4 | 7.9 | 8.5 | Yes | | No | |  |
| 30 | 233 | 4.1 | 19 | 12 | 300 | 41 | 0.6 | 6.4 | 1.86 | 22.1 | 107.9 | 0 | 0.53 | 10.5 | 12.9 | 9.8 | 3.6 | 16.5 | -0.9 | 8.8 | No | | No | |  |
| 31 | 129 | 3.6 | 24 | 18 | 151 | 31 | 1.2 | 5.1 | 0.94 | 49.1 | 91 | 0 | 0.15 | 3.7 | -8.1 | -4.7 | 7.2 | -0.9 | 5.1 | 0.3 | Yes | | Yes | |  |
| 32 | 238 | 3.7 | 12 | 7 | 150 | 22 | 0.4 | 6.8 | 1.41 | 46.2 | 112.9 | 0 | 0.42 | 4.7 | 3.1 | 20.7 | -1.1 | 2.0 | -9.2 | 11.5 | No | | No | |  |
| 33 | 216 | 4 | 14 | 15 | 124 | 20 | 0.6 | 9.3 | 1.88 | 34 | 91.5 | 0 | 0.05 | 3.3 | 7.0 | 22.5 | -3.9 | 3.1 | -12.8 | 9.7 | No | | No | |  |
| 34 | 228 | 3.5 | 17 | 15 | 150 | 16 | 1.1 | 5.4 | 0.83 | 59.7 | 100.4 | 0 | 0.03 | 3.2 | 7.1 | -18.8 | -1.3 | 5.8 | 19.8 | 1.0 | No | | Yes | |  |
| 35 | 210 | 3.4 | 15 | 26 | 163 | 18 | 1 | 7.7 | 1.08 | 60.2 | 104.8 | 0 | 0.01 | 3.9 | -5.4 | 1.0 | 4.5 | -0.9 | 1.7 | 2.7 | Yes | | No | |  |
| 36 | 200 | 4 | 18 | 19 | 154 | 50 | 0.9 | 7.9 | 1.19 | 53.4 | 111.2 | 0 | 0.03 | 2.3 | 5.1 | -1.0 | -6.4 | -1.3 | 2.2 | 1.3 | No | | Yes | |  |
| 37 | 224 | 3.6 | 18 | 33 | 253 | 56 | 0.8 | 7.9 | 1.23 | 51.6 | 108.5 | 0 | 0.25 | 4.2 | 0.5 | 15.7 | -1.5 | -1.0 | -10.4 | 5.3 | No | | No | |  |
| 38 | 302 | 3.6 | 11 | 5 | 272 | 9 | 0.8 | 5.1 | 1.1 | 37.5 | 112.5 | 0 | 0.08 | 2.3 | 1.0 | 10.9 | 0.1 | 1.1 | 14.1 | 25.0 | No | | No | |  |
| 39 | 510 | 3.2 | 20 | 18 | 481 | 41.8 | 0.5 | 6 | 1.45 | 38.4 | 95.5 | 0 | 0.19 | 12.5 | -1.8 | -39.3 | 7.2 | 5.3 | 46.6 | 7.4 | Yes | | Yes | |  |
| 40 | 208 | 4.2 | 20 | 12 | 110 | 18 | 0.7 | 7.4 | 1.23 | 36.2 | 113.1 | 1 | 0.26 | 8.1 | -5.3 | 3.7 | 0.4 | -5.0 | 0.2 | 3.9 | Yes | | No | |  |
| 41 | 200 | 3.6 | 18 | 21 | 256 | 42 | 0.7 | 6.6 | 1.53 | 41.7 | 115.1 | 0 | 0.02 | 4.8 | 3.2 | -9.2 | 5.1 | 8.3 | 15.0 | 5.9 | No | | Yes | |  |
| 42 | 253 | 3.2 | 13 | 9 | 182 | 20 | 0.6 | 4.3 | 0.95 | 51.5 | 91.5 | 0 | 0.16 | 8.7 | 11.4 | -2.7 | 1.6 | 13.0 | 10.7 | 7.9 | No | | Yes | |  |
| 43 | 136 | 4.2 | 24 | 18 | 204 | 49 | 0.6 | 6.1 | 1.03 | 45.4 | 98.9 | 0 | 0.14 | 3.4 | 3.6 | 8.2 | 5.2 | 8.9 | -4.0 | 4.3 | No | | No | |  |
| 44 | 173 | 3.4 | 13 | 9 | 127 | 24 | 0.5 | 6 | 0.94 | 49.1 | 94.6 | 0 | 0.08 | 3 | 6.4 | -4.5 | 3.6 | 9.9 | 7.3 | 2.9 | No | | Yes | |  |
| 45 | 229 | 3.7 | 30 | 29 | 310 | 51 | 0.5 | 4.8 | 1.11 | 53.7 | 102.9 | 0 | 0.06 | 5.6 | -0.9 | -1.4 | 1.7 | 0.9 | 8.6 | 7.2 | Yes | | Yes | |  |
| 46 | 184 | 4.5 | 19 | 11 | 218 | 29 | 0.5 | 8 | 1.34 | 31.4 | 102.3 | 0 | 0.08 | 3.8 | -5.0 | 0.8 | 2.3 | -2.7 | 3.8 | 4.6 | Yes | | No | |  |
| 47 | 164 | 4.7 | 19 | 23 | 201 | 18 | 1.4 | 5.4 | 0.73 | 101.2 | 89.7 | 0 | 0.01 | 3.6 | -13.6 | 0.8 | 12.7 | -0.8 | 6.8 | 7.6 |  | |  | |  |
| 48 | 234 | 4.3 | 35 | 40 | 291 | 16 | 0.5 | 7 | 1.01 | 69.7 | NA | 0 | 0.03 | NA | -5.9 | 3.9 | 0.6 | -5.3 | 4.1 | 8.0 |  | |  | |  |
| 49 | 442 | 3.8 | 20 | 15 | 169 | 44 | 0.5 | 2.6 | 0.62 | 105.7 | 98.6 | 0 | 0.16 | 4.5 | 2.2 | -7.6 | 15.6 | 17.8 | 0.8 | -6.8 |  | |  | |  |
| 50 | 260 | 5 | 21 | 13 | 134 | 27 | 0.8 | 7.7 | 0.99 | 70.7 | 92 | 0 | 0.03 | NA | 6.4 | -0.7 | -4.9 | 1.5 | 4.1 | 3.4 |  | |  | |  |
| 51 | 184 | 4.6 | 15 | 11 | 146 | 14 | 0.9 | 4.8 | 0.58 | 93.8 | 92.6 | 0 | 0.05 | 4.8 | 0.2 | -2.3 | 0.9 | 1.1 | 2.4 | 0.1 |  | |  | |  |
| 52 | 351 | 4.1 | 18 | 15 | 107 | 24 | 0.9 | 3 | 0.57 | 126.3 | NA | 1 | 0.14 | 8.1 | 5.6 | -1.1 | -1.7 | 3.8 | 2.0 | 0.8 |  | |  | |  |
| 53 | 282 | 4 | 17 | 18 | 208 | 34 | 0.6 | 5.6 | 0.75 | 92.2 | NA | 0 | 0.05 | NA | 3.9 | 0.7 | -0.1 | 3.7 | 2.5 | 3.1 |  | |  | |  |
| 54 | 229 | 4.7 | 29 | 26 | 121 | 47 | 0.8 | 8.2 | 1.01 | 66.1 | 121.2 | 0 | 0.02 | 2 | -12.9 | 11.7 | 9.7 | -3.2 | -5.8 | 5.9 |  | |  | |  |
| 55 | 178 | 4 | 18 | 12 | 34 | 153 | NA | 4.6 | 0.82 | 61.4 | NA | 0 | 0.12 | NA | -4.0 | 0.8 | 5.4 | 1.4 | 6.6 | 7.3 |  | |  | |  |
| 56 | 239 | 4.6 | 28 | 23 | 154 | 45 | 0.7 | 6.4 | 0.84 | 79.2 | 117 | 0 | 0.07 | 2.3 | -15.6 | 23.4 | 14.6 | -0.9 | 1.9 | 25.3 |  | |  | |  |
| 57 | 187 | 4.1 | 24 | 27 | 117 | 21 | 1 | 4.5 | 1.08 | 56.5 | NA | 0 | 0.08 | 3.8 | 4.0 | 2.4 | -2.9 | 1.0 | 2.6 | 5.0 |  | |  | |  |
| 58 | 244 | 4.2 | 24 | 19 | 245 | 33 | 0.6 | 5.6 | 0.85 | 52.1 | 98 | 0 | 0.03 | NA | -4.2 | 1.8 | 1.1 | -3.1 | 5.7 | 7.4 |  | |  | |  |
| 59 | 340 | 3.6 | 18 | 11 | 211 | 24 | 0.4 | 4.4 | 0.64 | 70.8 | NA | 0 | 0.05 | 4.9 | 2.2 | 5.3 | -0.9 | 1.3 | 0.3 | 5.6 |  | |  | |  |
| 60 | 209 | 4.6 | 18 | 16 | 263 | 44 | 0.8 | 8.4 | 1.24 | 46.3 | 106 | 0 | 0.17 | NA | 1.7 | -1.4 | -3.8 | -2.0 | 6.7 | 5.3 |  | |  | |  |
| 61 | 172 | 4.6 | 27 | 12 | 186 | 20 | 0.6 | 4.5 | 1.01 | 41.6 | 102.4 | 1 | 0.25 | NA | 16.6 | -3.5 | -4.4 | 12.2 | 2.8 | -0.8 |  | |  | |  |
| 62 | 175 | 4.3 | 16 | 12 | 210 | 22 | 0.7 | 7 | 1.2 | 46.2 | NA | 0 | 0.06 | 6.3 | 6.8 | -1.7 | -1.1 | 5.7 | 3.7 | 2.0 |  | |  | |  |
| 63 | 203 | 3.3 | 18 | 14 | 301 | 39 | 0.5 | 7.5 | 1.62 | 26.1 | 97.8 | 0 | 0.05 | 8.1 | -1.8 | 2.5 | 4.8 | 3.0 | 7.2 | 9.7 | Yes | | No | |  |
| 64 | 178 | 3.8 | 16 | 13 | 322 | 40 | 0.5 | 7.7 | 2.07 | 19 | 75.6 | 1 | 3.56 | 32.7 | 1.6 | -33.8 | 2.9 | 4.5 | 26.7 | -7.1 | No | | Yes | |  |
| 65 | 216 | 3.6 | 16 | 9 | 280 | 75.3 | 0.7 | 4.9 | 0.66 | 67.2 | 89.5 | 0 | 0.06 | 11.1 | -7.8 | -6.5 | 2.7 | -5.0 | 20.1 | 13.6 | Yes | | Yes | |  |
| 66 | 184 | 3.7 | 26 | 13 | 168 | 62 | 0.8 | 4.7 | 1.71 | 25.6 | 75.8 | 0 | 0.08 | 8 | 12.0 | 16.1 | -1.7 | 10.2 | 19.4 | 35.6 | No | | No | |  |
| 67 | 143 | 3.5 | 13 | 10 | 308 | 50 | 1.1 | 4 | 0.67 | 66.2 | 81.9 | 0 | 0.16 | 7.6 | -7.3 | -8.4 | 7.9 | 0.5 | 10.2 | 1.7 | Yes | | Yes | |  |
| 68 | 218 | 3.8 | 20 | 18 | 330 | 18 | 0.6 | 5.9 | 1.05 | 52.7 | 94 | 0 | 0.21 | 5.3 | -5.2 | -5.7 | -2.9 | -8.1 | 4.9 | -0.9 | Yes | | Yes | |  |
| 69 | 155 | 3.3 | 10 | 7 | 211 | 32 | 0.5 | 5.4 | 0.79 | 62.2 | 98.6 | 0 | 0.03 | 2.9 | -5.8 | -13.1 | 8.2 | 2.4 | 10.3 | -2.8 | Yes | | Yes | |  |
| 70 | 106 | 3.5 | 24 | 20 | 356 | 96 | 0.9 | 8.1 | 1.61 | 26.2 | 75.8 | 0 | 0.16 | 8.8 | -5.5 | 6.9 | 4.5 | -1.1 | -3.7 | 3.3 | Yes | | No | |  |
| 71 | 156 | 3.5 | 11 | 3 | 174 | 25 | 0.5 | 5.8 | 0.72 | 72.9 | 64.5 | 0 | 0.05 | 3.7 | -7.7 | -8.6 | 9.8 | 2.1 | 12.8 | 4.2 | Yes | | Yes | |  |
| 72 | 210 | 3.8 | 16 | 18 | 131 | 38 | 0.7 | 5.2 | 0.76 | 64.5 | 103.2 | 0 | 0.16 | 8.9 | 5.9 | 20.6 | -1.2 | 4.7 | 0.4 | 21.1 | No | | No | |  |
| 73 | 195 | 2.9 | 15 | 11 | 229 | 181 | 1 | 6.1 | 0.97 | 68.2 | 82.9 | 0 | 0.09 | 5.3 | 3.3 | 17.2 | 4.2 | 7.6 | 10.3 | 27.5 | No | | No | |  |
| 74 | 230 | 4.3 | 21 | 23 | 164 | 37 | 0.7 | 3.9 | 0.74 | 66 | 92 | 0 | 0.09 | 2.2 | 2.7 | -9.9 | 4.9 | 7.6 | 6.5 | -3.4 | No | | Yes | |  |
| 75 | 326 | 4.1 | 12 | 10 | 244 | 87 | 0.5 | 5.4 | 0.72 | 93.2 | 81.9 | 0 | 0.05 | 5.1 | -11.4 | -0.4 | 8.4 | -3.0 | 5.9 | 5.5 | Yes | | Yes | |  |
| 76 | 173 | 3.7 | 18 | 9 | 173 | 15 | 1.8 | 4.1 | 0.84 | 55.3 | 98.1 | 0 | 0.06 | 2 | 2.9 | -1.7 | -1.1 | 1.8 | 5.4 | 3.7 | No | | Yes | |  |
| 77 | 209 | 3.5 | 31 | 34 | 632 | 262 | 0.6 | 5.9 | 0.54 | 92.6 | 96.1 | 0 | 0.04 | 2.7 | -5.2 | 0.1 | 1.5 | -3.7 | 3.0 | 3.1 | Yes | | No | |  |
| 78 | 251 | 3.6 | 15 | 10 | 320 | 74 | 0.5 | 5.4 | 0.74 | 64.1 | 103.7 | 1 | 0.08 | 6.2 | -1.6 | -7.1 | -3.5 | -5.1 | 9.6 | 2.4 | Yes | | Yes | |  |
| 79 | 243 | 3.8 | 21 | 12 | 231 | 76 | 0.6 | 4.6 | 0.98 | 46.4 | 75.5 | 0 | 0.05 | 5.3 | -8.5 | 6.8 | 6.2 | -2.3 | -3.2 | 3.6 | Yes | | No | |  |
| 80 | 213 | 3.8 | 21 | 14 | 235 | 63 | 1.4 | 5.9 | 0.66 | 71.5 | 85.5 | 0 | 0.23 | 4.9 | 16.7 | 3.2 | -14.9 | 1.8 | -4.2 | -1.0 | No | | No | |  |
| 81 | 256 | 3.7 | 32 | 20 | 520 | 57.1 | 0.8 | 7.2 | 0.77 | 58.8 | 77 | 0 | 0.06 | 13.1 | -7.6 | -5.9 | 2.8 | -4.8 | 11.1 | 5.2 | Yes | | Yes | |  |
| 82 | 162 | 3.5 | 14 | 11 | 215 | 99 | 0.5 | 6.2 | 1.05 | 45.6 | 75.8 | 0 | 0.1 | 4.8 | 32.3 | 15.1 | 15.2 | 47.5 | -3.1 | 12.0 | No | | No | |  |
| 83 | 275 | 3.1 | 14 | 7 | 268 | 48 | 0.7 | 4.3 | 0.89 | 54.6 | 77 | 1 | 0.09 | 6.1 | 12.4 | 7.2 | -7.0 | 5.4 | 4.7 | 12.0 | No | | No | |  |
| 84 | 225 | 3.5 | 14 | 8 | 170 | 32 | 0.4 | 7.1 | 1.25 | 36.6 | 83 | 1 | 0.06 | 4.4 | -3.2 | 50.9 | 18.7 | 15.5 | 3.9 | 54.7 | Yes | | No | |  |
| 85 | 134 | 3.8 | 25 | 20 | 204 | 67 | 0.8 | 4.5 | 0.62 | 78.7 | 95.5 | 1 | 0.13 | 4.2 | 0.0 | 5.6 | 1.6 | 1.5 | -4.5 | 1.1 | Yes | | No | |  |
| 86 | 169 | 3.7 | 15 | 12 | 372 | 86 | 0.8 | 7.6 | 1.28 | 38.6 | 65.5 | 0 | 0.87 | 3.4 | -3.0 | -41.9 | 0.8 | -2.1 | 4.3 | -37.7 | Yes | | Yes | |  |
| 87 | 131 | 3.5 | 26 | 26 | 339 | 91 | 0.7 | 6 | 1.14 | 40 | 74.7 | 0 | 0.05 | 3.8 | 11.6 | NA | -11.7 | 0.0 | NA | NA | No | | NA | |  |
| 88 | 156 | 3.2 | 16 | 8 | 240 | 51 | 0.7 | 6.3 | 0.89 | 51.6 | 91.5 | 0 | 0.05 | 2.7 | -35.0 | -5.9 | 27.9 | -7.1 | 32.0 | 26.2 | Yes | | Yes | |  |
| 89 | 193 | 3.1 | 18 | 10 | 325 | 6 | 0.5 | 8.8 | 1.66 | 35.6 | 100.4 | 0 | 0.1 | 5.2 | -9.0 | 0.9 | 4.7 | -4.3 | 5.3 | 6.1 | Yes | | No | |  |
| 90 | 147 | 3.9 | 16 | 15 | 253 | 36 | 0.7 | 5.9 | 0.99 | 42.2 | 75 | 0 | 0.11 | 5.8 | 54.2 | 3.1 | -55.2 | -1.0 | 0.7 | 3.8 | No | | No | |  |
| 91 | 88 | 3.6 | 23 | 14 | 315 | 73 | 0.6 | 7 | 1.5 | 28.1 | 89.6 | 0 | 0.08 | 4.8 | -3.6 | -4.4 | 3.7 | 0.1 | 6.9 | 2.5 | Yes | | Yes | |  |
| 92 | 145 | 2.8 | 65 | 43 | 636 | 87 | 3.3 | 5.2 | 0.59 | 77.4 | 91 | 0 | 0 | NA | -0.7 | 8.1 | 6.6 | 5.9 | 2.6 | 10.7 | Yes | | No | |  |
| 93 | 184 | 3.6 | 17 | 13 | 162 | 32 | 0.6 | 4.1 | 0.6 | 86.4 | 79.5 | 0 | 0.04 | 3.4 | -19.2 | 0.4 | 21.9 | 2.8 | 9.5 | 9.9 | Yes | | No | |  |
| 94 | 164 | 3.7 | 14 | 6 | 249 | 61 | 0.3 | 4 | 0.82 | 59.4 | 95.5 | 0 | 0.24 | 7.4 | -9.7 | 0.6 | 16.0 | 6.3 | -1.2 | -0.6 | Yes | | No | |  |
| 95 | 124 | 3.2 | 23 | 15 | 307 | 54 | 1 | 7.5 | 0.97 | 47.2 | 78.1 | 1 | 0.11 | 10.3 | -1.1 | 2.8 | -0.6 | -1.7 | 9.2 | 12.0 | Yes | | No | |  |
| 96 | 137 | 3.7 | 23 | 16 | 181 | 38 | 0.7 | 8.7 | 1.14 | 38.7 | 84.4 | 0 | 0.3 | 4.3 | -0.7 | -2.3 | -1.4 | -2.2 | 2.3 | 0.0 | Yes | | Yes | |  |
| 97 | 146 | 4.1 | 20 | 19 | 322 | 95 | 0.7 | 5.6 | 1.21 | 35.1 | 85.3 | 0 | 0.24 | 5.3 | -4.1 | -4.7 | 2.1 | -2.0 | 4.5 | -0.2 | Yes | | Yes | |  |
| 98 | 223 | 3.3 | 27 | 29 | 416 | 71 | 0.6 | 7.6 | 2.34 | 16.9 | 77 | 1 | 0.62 | 9.5 | 5.1 | NA | 0.8 | 5.9 | NA | 80.7 | No | | NA | |  |
| 99 | 177 | 4 | 21 | 16 | 507 | 202 | 0.8 | 4.4 | 0.65 | 79.7 | NA | 0 | 0.1 | NA | -1.8 | -0.8 | 15.0 | 13.3 | 9.6 | 8.8 | Yes | | Yes | |  |
| 100 | 287 | 3.3 | 23 | 15 | 433 | 33 | 0.6 | 4.1 | 0.81 | 61.8 | NA | 0 | 0.06 | NA | 0.6 | -4.3 | 8.7 | 9.4 | 15.2 | 10.9 |  | |  | |  |
| 101 | 175 | 4 | 15 | 112 | 262 | 90 | 0.8 | 3.7 | 0.69 | 71.3 | NA | 1 | 0.19 | 4.2 | -4.5 | -29.5 | -0.4 | -4.9 | 22.4 | -7.0 |  | |  | |  |
| 102 | 308 | 3.9 | 17 | 10 | 358 | 131 | 0.9 | 4.3 | 0.87 | 55.3 | 80 | 0 | 0.11 | NA | 11.4 | 0.5 | 16.7 | 28.1 | 3.7 | 4.2 |  | |  | |  |
| 103 | 260 | 3.6 | 13 | 11 | 236 | 75 | 0.6 | 7.5 | 1.5 | 30.3 | 74.5 | 1 | 0.16 | 7.8 | 18.7 | -7.7 | -16.4 | 2.3 | 9.2 | 1.6 |  | |  | |  |
| 104 | 252 | 3.9 | 13 | 9 | 76 | 25 | 0.5 | 6.8 | 1.11 | 41.6 | 77.7 | 0 | 0.08 | 8.1 | 1.8 | -2.4 | 0.0 | 1.8 | 4.7 | 2.3 |  | |  | |  |
| 105 | 171 | 4.3 | 22 | 24 | 225 | 136 | 0.5 | 7.1 | 0.9 | 65.7 | NA | 0 | 0.1 | 6.3 | -8.1 | -11.7 | 2.0 | -6.1 | 13.8 | 2.1 |  | |  | |  |
| 106 | 263 | 3.6 | 25 | 10 | 426 | 85 | 0.9 | 6.5 | 1.23 | 34.3 | 81.7 | 1 | 0.14 | 16.9 | 4.7 | -55.0 | 9.0 | 13.7 | 61.8 | 6.9 |  | |  | |  |
| 107 | 166 | 3.6 | 16 | 13 | 248 | 53 | 1.1 | 5.5 | 1.49 | 27.3 | 1 | 0 | 1.03 | NA | -1.9 | -6.7 | 2.1 | 0.3 | 11.7 | 5.0 |  | |  | |  |

Abbreviation: Plt; platelet count, Alb; albumin, AST; aspartate transaminase, ALT; alanine transaminase, ALP; alkaline phosphatase, γGTP; gamma-glutamyl transpeptidase, bil; total bilirubin, UA; uric acid, Cre; creatinine, eGFR; estimated glomerular filtration rate, PT; prothrombin time test, NAG; N-acetyl-beta-D glucoaminidase, NA; not available.
