# Supplementary material for: Potential effect of tolvaptan on polycystic liver disease for patients with ADPKD meeting the Japanese criteria of tolvaptan use
Source: PLoS One. 2022 Feb 17;17(2):e0264065. doi: 10.1371/journal.pone.0264065 (PMC8853523; doi:10.1371/journal.pone.0264065)
Supplement: S1 Fig — To assess the dose effect of tolvaptan on the change in annual growth rate of total liver volume (TLV), the original tolvaptan group were stratified by daily dose of tolvaptan, less than 30mg, 60mg, 90mg, and mg. However, we could not find the any trends of change in annual growth rate of total liver volume nor response rate associated to the dose of tolvaptan. Abbreviation.ΔTLV% change in annual growth rate of TLV. (PDF) [file pone.0264065.s001.pdf]

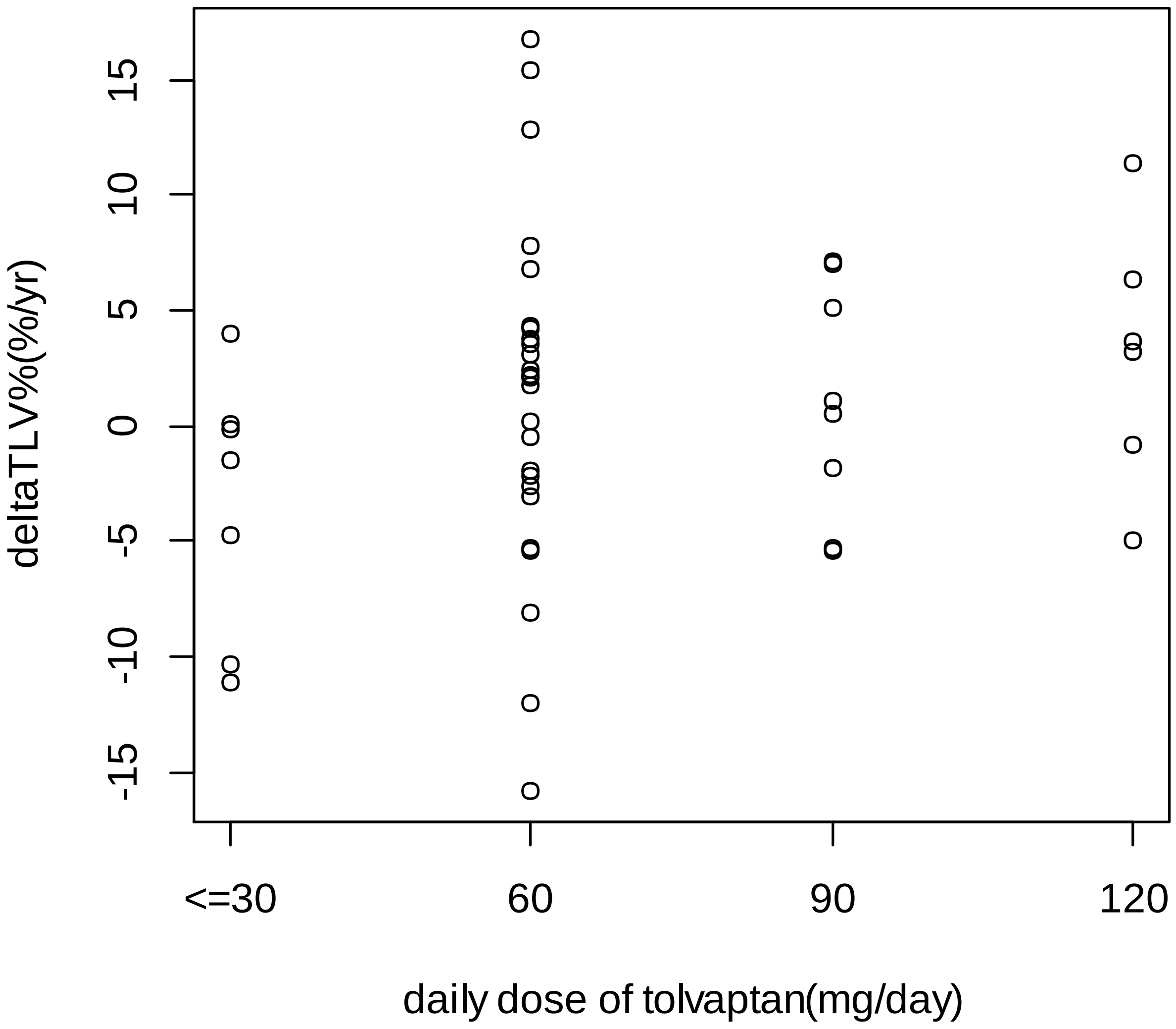
deltaTLV%(%/yr)
15
10
5
0
-5
-10
-15
<=30
60
90
120
daily dose of tolvaptan(mg/day)

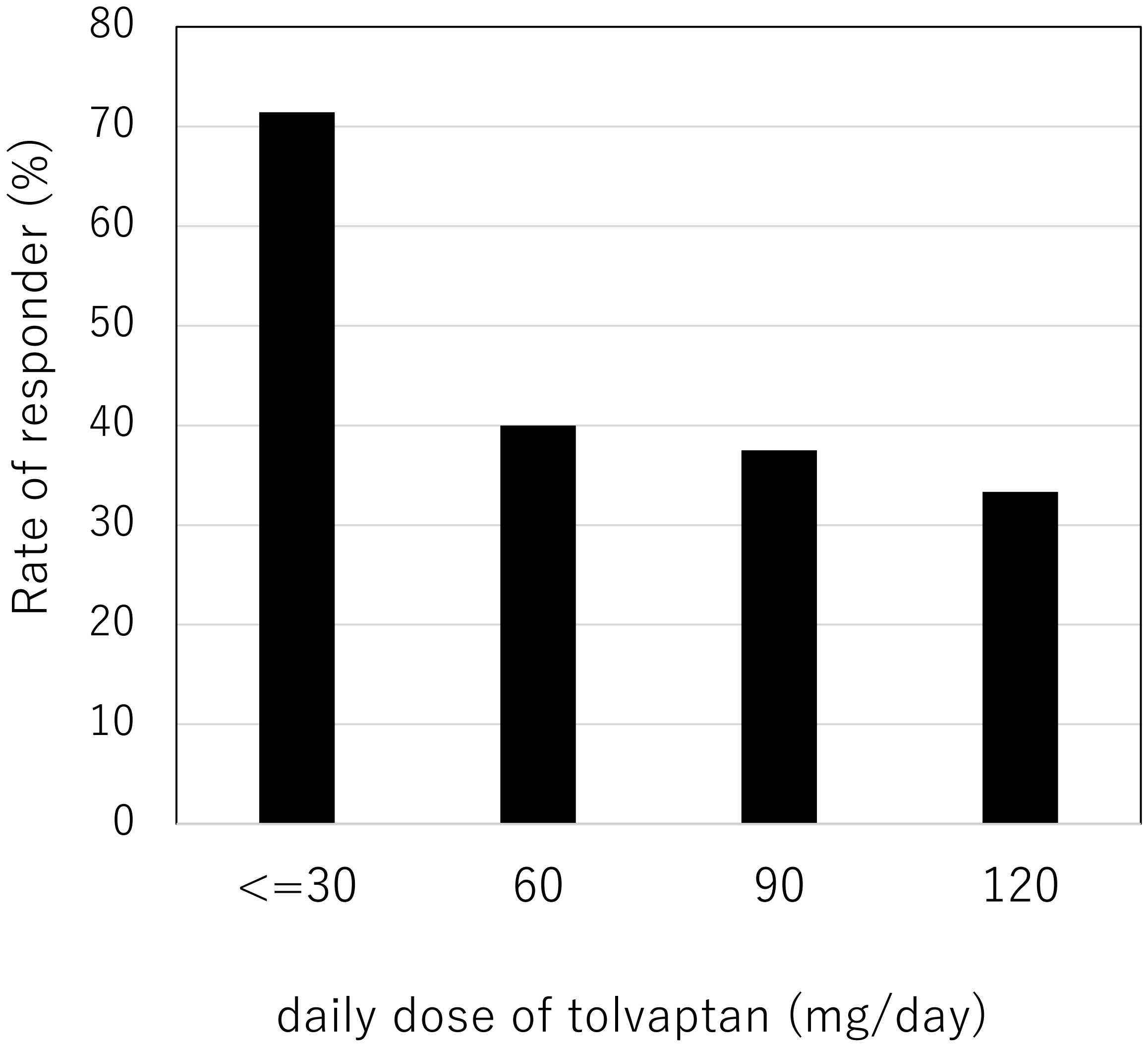
Rate of responder (%)
80
70
60
50
40
30
20
10
0
<=30
60
90
120
daily dose of tolvaptan (mg/day)
