# Supplementary material for: Potential effect of tolvaptan on polycystic liver disease for patients with ADPKD meeting the Japanese criteria of tolvaptan use
Source: PLoS One. 2022 Feb 17;17(2):e0264065. doi: 10.1371/journal.pone.0264065 (PMC8853523; doi:10.1371/journal.pone.0264065)
Supplement: Attachment — Submitted filename: 20210726 letter to editor and rebuttal letter.docx [file pone.0264065.s009.docx]

Dear Editor and Reviewers

Thank you for reviewing our manuscript “Potential effect of tolvaptan on polycystic liver disease with ADPKD [PONE-D-21-12669]” and offering valuable advices during the tough situation by COVID19 pandemic. We also appreciate the time and effort you and each of the reviews have dedicated to providing insightful feed back on our paper.

In addition, thanks to your generous kindness of extending the deadline of resubmission, we have finished answering all the comments. The revised manuscript would be improved over the initial manuscript.

We have addressed your comments with point-by-point responses and revised the manuscript accordingly. We would like to send two versions, clean and marked one and as well as the ppt file of graphic abstract.

Response to Editor and Reviewers’ comments

Editor:

1. *Please ensure that your manuscript meets PLOS ONE's style requirements, including those for file naming. The PLOS ONE style templates can be found at*

*https://journals.plos.org/plosone/s/file?id=wjVg/PLOSOne_formatting_sample_main_body.pdf*

[*https://journals.plos.org/plosone/s/file?id=ba62/PLOSOne_formatting_sample_title_authors_affiliations.pdf*](https://journals.plos.org/plosone/s/file?id=ba62/PLOSOne_formatting_sample_title_authors_affiliations.pdf)

RESPONSE: We had changed all the file naming according to PLOS ONE’s style requirements.

2. *In your ethics statement in the manuscript and in the online submission form, please ensure that you have discussed whether all data/samples were fully anonymized before you accessed them and/or whether the IRB or ethics committee waived the requirement for informed consent. If patients provided informed written consent to have data/samples from their medical records used in research, please include this information.*

RESPONSE: We had added the expression describing fully anonymized before we accessed data on page 7 line 17 and the sentence on page 8 line 1-2.

*3. Please include the following information in your Methods:*

*1) Whether the use of tolvaptan was implemented as standard-of-care at your hospital throughout the study period*

RESPONSE: In this retrospective observational study, tolvaptan was used standard-of-care. We have added the following sentence “The use of tolvaptan was implemented as standard-of care throughout the study period” on Page 7 Line 14-15.

2) *Whether assignment of patients to tolvaptan was made at the discretion of the treating physician or for the purposes of research.*

RESPONSE: In this retrospective observational study, tolvaptan was prescribed by the treating physician not by the purpose of research. We have added the following sentence “the assignment of patients to tolvaptan was made at the discretion of the treating physician”, on Page 7 Line 16.

4. *Please note that PLOS does not permit references to 'data not shown.' Authors should provide the relevant data within the manuscript, the Supporting Information files, or in a public repository. If the data are not a core part of the research study being presented, we ask that authors remove any references to these data.*

RESPONSE: After conducting the revising analysis, we considered that this information is not a core part, therefore, we decline the expression ‘data not shown’, on Page 25 Line 13.

5. *Financial Disclosure section:*

*"**This work was supported by J.H’s competitive research grant from Otsuka Pharm, Japan, and a research grant from the Okinaka Memorial Institute for Medical Research, and H.M’s research grant from Toranomon Hospital. The funders had no role in study design, data collection and analysis, decision to publish, or preparation of the manuscript."*

*We note that you received funding from a commercial source: Otsuka Pharm*

*Please provide an amended Competing Interests Statement that explicitly states this commercial funder, along with any other relevant declarations relating to employment, consultancy, patents, products in development, marketed products, etc.*

*Within this Competing Interests Statement, please confirm that this does not alter your adherence to all PLOS ONE policies on sharing data and materials by including the following statement: "This does not alter our adherence to PLOS ONE policies on sharing data and materials.” (as detailed online in our guide for authors http://journals.plos.org/plosone/s/competing-interests). If there are restrictions on sharing of data and/or materials, please state these. Please note that we cannot proceed with consideration of your article until this information has been declared.*

*Please include your amended Competing Interests Statement within your cover letter. We will change the online submission form on your behalf.*

*Please know it is PLOS ONE policy for corresponding authors to declare, on behalf of all authors, all potential competing interests for the purposes of transparency. PLOS defines a competing interest as anything that interferes with, or could reasonably be perceived as interfering with, the full and objective presentation, peer review, editorial decision-making, or publication of research or non-research articles submitted to one of the journals. Competing interests can be financial or non-financial, professional, or personal. Competing interests can arise in relationship to an organization or another person. Please follow this link to our website for more details on competing interests: http://journals.plos.org/plosone/s/competing-interests*

RESPONSE: We have amended Competing Interests Statement on cover letter and we have added the sentence you offered.

Reviewer #1:

*The analysis is simple but appropriate for the conducted analysis.*

*However, the figures 2-4 look strange. I do not know what information they should provide nor is it explained in the paper. The figures are not related to the numerical analysis but provide a visual overview, which is good to provide. They are just unclear! Please discuss them in detail.*

RESPONSE:

Thank you for pointing out figure 2-4’s roles played on this article. After re consideration during revising process, data of figure 2 and figure 4 did not directly related to the major conclusion on this article. Therefore, we declined resubmitting figure 2 and figure 4. Whereas, since figure 3 was the primary outcome of this study, we decided to keep using it, then the figure number was changed from figure 3 to figure 4 in the revised manuscript. In addition, we added the figure caption on Page 17 Line 12.

*Reviewer #2:*

Thank you for providing us with the suggestive comment on our article. We totally agreed with your comment 1, the necessity of the control group. Although it took an enormous time to find the appropriate control group after screening 902 patients, we found 25 patients who were eligible to the control group. Compared with this control group, we could not find the statistical difference of the change in the annual growth rate of TLV between groups. However, we hope this revising process would strengthen our article and give more meaningful suggestions to the future study.

In order to answer the all the valuable comments from editors and reviews, we decided to decline figure 2 and 4 of the initial manuscript, then we added 2 figures (figure 2 and figure3) and supporting information including 4 figures and 3 tables. Figure 3 of the initial manuscript left as figure 4.

*1. Although there were 902 patients with PLD or PKD in total, less than 10% were analyzed in this study, which may constitute a limitation. Given that there were more than 600 patients who did not take tolvaptan, authors might want to calculate the growth rate of TLV in those patients with CT scans available, and compare the rate with the 82 eligible patients. This analysis would make the study more comprehensive, even if the growth rate is not significantly altered.*

RESPONSE: We agreed with the statistical limitation of this original manuscript of single arm observational study. Therefore, we decided to find the control group that would be compared to the tolvaptan group. After screening 902 patients to find the appropriate control group, defined ADPKD patients with polycystic liver and fulfilled Japanese criteria of tolvaptan use, and had more than three times CT scanning. We finally found 25 patients that met the prescription criteria of tolvaptan in Japan and set it as a control group. Compared to this control group, the tolvaptan group were larger height adjusted TKV, higher serum creatinine level, and lower eGFR. We divided the observational period of the control group and compared the difference of annual liver growth rate, but there was no statistical difference.

We added the method of this analysis on Page 6, Line 15-17, and the result of this analysis from page 15 line 6 to page 16 line 7 including figure 2 of revised manuscript.

We hope these results will make this article have more statistical robustness.

*2. The authors showed that the median growth rate of TLV did not change after taking tolvaptan. However, it is unclear whether the study participants did show response to tolvaptan in terms of growth rate of TKV.*

RESPONSE: Thank you for giving us the important advice to evaluate the effect of tolvaptan on polycystic kidney. The annual change in TKV of the tolvaptan group was 0.2 (-42.0, 50.9) %/year while that of the control group was and -1.1 (-55.0, 23.4) %/year. The effect of tolvaptan on the growth rate of TKV was neither significant in this study. We thought that this fact would be partially derived from the liver-dominant ADPKD and less progressive ADPKD patients were selected in this study. We added this result from page 15 line 16 to page 16 line 1 and on figure 3 as well as this limitation on page 25 line 17.

*3. The maintenance dose of tolvaptan in each group was not described in Table 1. Was the dosage not associated with the response in PLD?*

RESPONSE: We added the dose of tolvaptan in Table1 and Table2. We created the four categories according to the dose of tolvaptan, however, there was no trend that suggested the dose effect of tolvaptan on response rate or decline of liver growth rate. We added the result of this subgroup analysis on page 17 line 8 and on Supporting Information Figure S1.

*4. Figures and figure legends need improvement.*

*- In Figure 2, there is no explanation what blue and red lines mean. This figure also needs the label (A or B) according to the text.*

RESPOND: Thank you for pointing out the insufficient contents of the legend. As mentioned above. During the revising process, however, we reconsider that the result of this figure was not directly related to the major conclusion of this study, therefore, we decided to decline this figure.

*- Order of Figure 3 and Figure 4 could be the other way around. In the main text, Figure 3 is supposed to show the association between TLV and TKV, whereas Figure 4 should show the delta TLV% before and after tolvaptan.*

*- Figure 4 (association between PLD and PKD): please add correlation coefficient and p value.*

*- Legends in each figure need more information so that the readers can understand without referring to the main text.*

RESPONSE:

Thank you for pointing out the insufficient contents of the legend. We added the figure legends on figure 4 (delta TLV% before and after tolvaptan) and added the statistical information into the figure. The result of figure3 (the association between TLV and TKV) was not directly related to the major conclusion of this study, so we decided to decline this figure,

*5. Page 8, line 3. aldosterone > angiotensin II*

RESPONSE: We have changed the word above (Page8, line 7).

*6. Page 9, line 16 (statistical analysis). Is the statistical analysis two-tailed or one-tailed? Please describe.*

RESPONSE: We conducted two-tailed Fisher’s exact test. We added the expression “two-tailed” in Method section on page 10 line 9.

*7. Page 17, line 15-16; consider revising the sentence. V2R is now known to be more widely expressed in the kidney, including thick ascending limb of Henle (Ref: PMID 32035616 and 17626156).*

RESPONSE: Thank you for showing us valuable reference articles. We adopted the sentence above from Page22, Line 18 to Page 23, Line 1 and added the reference articles.

*8. “Height adjust total liver volume” in figure legends could be “Height adjusted total liver volume”.*

RESPONSE: We have changed the expression in figure legends.

*Reviewer #3: Dear authors,*

*While the efficacy of Tolvaptan in reducing renal cyst growth has been established, its efficacy in complicating extrarenal cysts, particularly in more frequent hepatic cysts, is unknown at present. This study tried to analyze this point, and it is considered to be a informative study with significant clinical advantages, even if the result is positive or negative. However, there are several issues to be resolved as shown below.*

RESPONSE: Thank you for providing us the suggestive comment on our article.

After reconsidering the design of this study, subgroup analysis for the patients without the history of physical intervention were conducted to minimized their effect on PLD. We hope this revising data will strengthen our article and give more meaningful suggestion to the future study.

*Major*

*Comment #1*

*This study was a retrospective observational study design without a control group, and the analysis is restricted to individuals with ADPKD who meet the Japanese National criteria for tolvaptan use. Thus, the effect of tolvaptan on hepatic cysts in patients with less severe or less active PKDs or hepatic cysts in patients with predominant hepatic than renal cysts is not included in the analysis. It should be specified that this study is an analysis restricted to hepatic cysts in subjects with ADPKD who met the criteria for tolvaptan use, including title. Although the significance of this study is clearly understandable, I believe that it is inappropriate to refer directly to the effects of tolvaptan in restrospective single arm observation, not in randomized controlled trial, and that careful representation or description is desirable.*

RESPONSE: We agreed with your suggestion to change more suitable title for this study. In this revised manuscript, we conducted the analysis by using control group, however, several limitation still had existed and the patients included in this study was limited within the ADPKD patients who met the Japanese criteria of tolvaptan use. Therefore, we changed the title to “Potential effect of tolvaptan on polycystic liver disease for patients with ADPKD meeting the Japanese criteria of tolvaptan use”.

We hope this title would be more precisely described and more suitable to be accepted.

*Comment #2*

*Cases with or without hepatic cyst drainage or TAE are mixed in the study population.*

*In such cases with physical interventions, organic changes or damages may have been occurred in the pericystic tissues associated with invasions, and it is considered inappropriate to analyse populations that may differ qualitatively. Alternatively, it should be discussed that physical interventions are independent of the effects of cyst growth or tolvaptan. I would like to ask for the opinions of the authors. Even if the sample size is reduced, it is considered that the population should be restricted to patients without those physical interventions, or that the subgroup analyses should be added at the least.*

RESPONSE: We agree with subgroup analysis for the patients without the history of the physical intervention. After excluding the physical intervention, such as liver trans-arterial embolization and drainage for liver cyst, 46 patients were eligible for analysis. In this group, there was no significant difference of annual liver growth rate before and after the tolvaptan use, however, 20(43.4%) patients had experienced the reduction of liver growth rate, responder. Multivariable regression analysis adjusted related variables showed that age and baseline liver growth significantly predicted the responder. We deleted the carry-over effect of trans-arterial embolization of liver from the paragraph about limitation of this study in the discussion part, then we added these results as subgroup analysis from page 21, line 2 to page 22, line 8 and on Supporting Information.

We believe that these subgroup analysis will more efficiently exclude the carry-over effect of physical intervention and will make our result more clearly understandable.

.

*Comment #3*

*It is of great interest that responder, non-responder analysis showed that the more active PLDs, the more pronounced the cyst reduction after tolvaptan use, as well as their efficacy in renal cysts. The results strongly suggest that a similar mechanism to inhibition or reduction of renal cyst growth by tolvaptan may apply to hepatic cysts.*

*On the other hand, it is not well understandable that there was no correlation between kidney volume reduction and liver volume reduction in the responder group. The influence of the drainage cases and TAE cases may be concerned, but how do the authors consider this point?*

RESPONSE:

As we mentioned in the response to comment#2, we conducted subgroup analysis for the patients without the history of the physical intervention. Although the rate of the responder was reduced after excluding these interventions, the prognostic factors related to the responders were the same as that of the main analysis. The result of the main analysis contained such carry-over effects related to the physical intervention, however, we believe this well-designed subgroup analysis minimized this concerned effect.

*Comment #4*

*There is a lack of discussion of the impact of spontaneous shrinkage of hepatic cysts.*

*In Ref-8, as a consequence of a large observational study of PLDs, it is reported that spontaneous reduction of hepatic volume is observed in 30% of cases in the population. Ref-9 has also reported a reduction in hepatic volume in half of placebo group. In this study, the liver volume is reduced in about half of the subjects, but how to consider the involvement of spontaneous reduction should be discussed.*

RESPONSE: Thank you for pointing out this spontaneous effect. We totally agree that, in our study, the reduction of the annual growth rate of TKV was partially explained by this phenomenon because the patients of this study were older than previous studies. However, the logistic regression analysis adjusting related variables showed that higher growth rate of TLV as well as older age significantly predicted responders. Therefore, we consider that the reduction observed in our study would be not fully derived from the spontaneous reduction of PLD.

Although it was difficult to estimate the rate of spontaneous reduction, we added this problem from page 24 line 12 to page 25 line 1.

*Minor*

*Comment #1*

*In a stratified analysis of responder and non-responder, adjustments for variables have been described, however, specific method for this statistical procedure has not been described in the text.*

RESPONSE: We wrote down the method on Page 10 line 14-18.

*Comment #2*

*Inconsistencies are seen in figure description in the text and figure numbering (e.g. Fig. 2B in P14L7). In addition, both Fig. 2 and Fig. 4 are not represented in A and B.*

RESPONSE: Thank you for pointing out incorrect numbers of figure 2 and figure4. However, we decided to decline these figures in revised manuscript.

*Reviewer #4:*

RESPONSE: Thank you for providing us the suggestive comment on our article. Dose effect of tolvaptan and menopausal status were evaluated by conducting subgroup analysis.

In addition, we set the control group after screening 902 patients again secondary analysis was conducted for patients without the history of intervention to polycystic liver to minimized the carry-over effect of such therapies.

After reconsidering the values of figures, we decided to decline figure 2 and figure 3 because we considered that these figures did not directly represent the main result of this study.

We hope that these revisions would make more appropriate this study design.

*Polycystic liver disease leads to pain and suffering in many individuals with autosomal dominant polycystic kidney disease. This interesting study examines changes in total liver volume in a cohort of individuals who took tolvaptan and followed longitudinally. About 50% experienced a reduction in liver volume and about 50% experienced a continued increase in liver volume. The paper is well written. However I am concerned about the statistics.*

*There are number of extreme outlier cases who could have influenced the data analysis. Overall we may be seeing a regression to the mean.*

*Please mark individuals that had TAE or liver volume reducing procedures on the graph.*

RESPONSE: Thank you for the carry-over effect of the physical intervention of polycystic liver, such as liver trans-arterial embolization and liver cyst drainage. We decided to decline to use figure 2 and figure 4 in the revised manuscript however, we conducted the secondary analysis about patients without the history of physical intervention in this major revision.

We added this subgroup analysis from page 21, line2 to page 22, line 8 and on Supporting Information.

*Please add figure showing both females that were pre and post menopausal and describe what happened to their total liver volumes.*

RESPONSE:

We collected the data about menopausal status about female patients in the tolvaptan group. Among them, we found the status only from 44 female patients. From the subgroup analysis for the groups according to the menopausal status, however, the annual growth rate of TLV was not significantly different before and after tolvaptan use.

We added the result in Supporting Information and on page17 line 8-10.

*If there is a positive effect of tolvaptan on total liver volume then one might expect a dose -response effect. No dose information has been provided.*

RESPONSE: We conducted the subgroup analysis to evaluate the dose effect of tolvaptan. The tolvaptan group were divided into the four categories according to the maintenance dose of tolvaptan, however, there was no trend that suggested the dose effect of tolvaptan on response rate or decline of liver growth rate. We added the result of this subgroup analysis on page 17 line 8 and on Supporting Information Figure S1.

*******************************************************************************************

Again, thank you for giving us the valuable comments in order to strengthen our manuscript. For these seven weeks we have been trying hard to incorporate your feedback and we hope that these revisions persuade you to consider the acceptance of our submission.

Sincerely,

Hiroki Mizuno.

Nephrology Center

Toranomon Hospital Kajigaya, Japan

[hilomiz@yahoo.co.jp](mailto:hilomiz@yahoo.co.jp)

+08-44-3588-5111
