# Supplementary material for: Potential effect of tolvaptan on polycystic liver disease for patients with ADPKD meeting the Japanese criteria of tolvaptan use
Source: PLoS One. 2022 Feb 17;17(2):e0264065. doi: 10.1371/journal.pone.0264065 (PMC8853523; doi:10.1371/journal.pone.0264065)
Supplement: Attachment — Submitted filename: 20211209 letter to editor and rebuttal letter.docx [file pone.0264065.s010.docx]

Dear Editor and Reviewers

Thank you for reviewing our manuscript “Potential effect of tolvaptan on polycystic liver disease for patients with ADPKD meeting the Japanese criteria of tolvaptan use [PONE-D-21-12669R1]” and offering valuable advices. We also appreciate the time and effort you have dedicated to providing insightful feedback on our paper.

We have addressed to your comments with point-by-point responses and revised the manuscript accordingly. We would like to send two copies, clean and marked versions and as well as the pdf file of four figures.

Response to Editor and Reviewers’ comments

To Editor

Dear Editor

Thank you for reviewing our first manuscript.

In order to answer the reviewer#4’s suggestion, we decided to amend the whole part of the method and result of the first revision, because the patients with a history of physical interventions needed to be excluded in the main analysis. As a result, in this second revision, the number of patients in the cohort reduced from 107 to 62, however, we expect that this amendment would make our study clearer to understand the true effect of tolvaptan on PLD without bothering the carry-out effect of physical interventions of PLD.

It would be appreciated if you could review the new manuscript of the second revision.

Reviewer #2

Dear Reviewers

Thank you for reviewing our first manuscript.

In order to minimize the carry-out effect of physical interventions of PLD, which was pointed out by another reviewer, we excluded the patients having the previous history of such procedures including cyst drainage and trans-arterial embolization for liver arteries. In this second revision, however, the conclusion of the results was consistent with the first revision although the number of patients reduced from 107 to 62.

It would be grateful that you could review our manuscript in this new study design.

To Reviewer #3

*Dear Reviewer #3:*

Thank you for pointing out the valuable suggestion to strengthen our manuscript. As reviewer #4 pointed out, in this second revision, the inclusion criteria were modified in order to exclude the carry-out effect of the physical intervention of PLD. The number of eligible patients decreased from 107 to 62, however, we believe this revision would make our study more efficient to analyze the tolvaptan’s effect on PLD.

*“Thank you for responding to your opinion about the title. I believe that modification of the title has led to a more accurate representation of the study design. And, it is grateful to respond to the previous commentary that the case which carried out physical intervention should be analyzed as a subgroup.*

*In this revised version, total liver volume of patients administered tolvaptan was compared with that of patients who met the criteria of tolvaptan use in Japan and were not administered tolvaptan. However, this study has been conducted as a retrospective longitudinal observational study and is not a prospective randomized controlled trial.*

*Therefore, it is unlikely that patients who do not receive tolvaptan in this analysis are adequate as a control group because the selection bias of whether or not to use tolvaptan occurs when the treatment of choice was performed on the tolvaptan use. In addition, it is considered that it should be corrected to "non-tolvaptan" rather than “control”. However, it is favorable that the authors are trying to objectively assess the effectiveness of tolvaptan while there is a limitation in the retrospective study.”*

RESPONSE: We agree with your idea of describing “non-tolvaptan group” because tolvaptan was not intended to be prescribed for these patients. We have changed all the expression from “control group to “non-tolvaptan group”

*“However, it is problematic that the authors described "53.6% of ADPKD patients taking tolvaptan experienced the reduction of the annual growth rate of TLV" as a conclusion. The outcome of this revised version is that tolvaptan is not efficacious in reducing hepatic cysts. Therefore, it may be concluded from statistical analyses that the reduction in TLV seen in about half of tolvaptan users is not of particular significance. I would like to know the author’s opinion on this issue.”*

RESPONSE: As well as in the first revision, the primary analysis of the difference of annual growth rate of tolvaptan in this second revision was not significantly different between tolvaptan group and non-tolvaptan group. Therefore, we considered that the tolvaptan’s effect on the annual growth rate of TLV was not established in this retrospective observational study, and then, we decided to describe the conclusion as follows.

“the change in annual growth rate of TLV in ADPKD patients taking tolvaptan was not statistically different compared with that in ADPKD patients without taking tolvaptan. However, tolvaptan may have the potential to suppress the growth rate of TLV in some PLD patients due to ADPKD, especially in older patients or those that are rapid progressors of PLD.”

*“The following is my personal opinions, but it is generally known that the TKV-reducing or-suppressing effects of tolvaptan are deviated in individual case. The same situation may be true for TLV reduction, and there may be an analysis of TLV reduction limitedly in the subgroup showing responder on TKV.”*

RESPONSE: Thank you for giving us the valuable suggestions about the association between TKV responder and TLV responder. Although the dose effect of tolvaptan on TLV was not found in our study, and as you mentioned the individual variability of tolvaptan’s effect existed in our cohort, in this cohort of secondary revision, we found that the response rates of TLV in patients with responder on TKV (64.9%) was larger than that in non-responder on TKV (45.2 %) (p<0.01). In addition, the change of annual liver growth rate was mildly associated with the change of the annual kidney growth rate (p<0.01) although rho was relatively lower level (rho=0.34). These facts may imply that some molecular mechanism expressed in both liver and kidney, such as polycystin-1 might be related to the effect of tolvaptan on liver or kidney although the expression rate of polycystin-1 was not detected in our study.

*Minor*

*“Are the ∆TLV% described on the Y-axis in Fig. 2 and annual liver growth rate on the X-axis in Fig. 4 not identical? If the same, the representations should be unified.”*

RESPONSE: delta TLV% represents the difference of annual liver growth rate between observational period and tolvaptan period, while TLV% in Fig 4 represents the annual liver growth rate. We unified these expressions between figures.

*Reviewer #4:*

Dear Reviewer #4

Thank you for reviewing the manuscript of our first revision and giving us valuable suggestions to improve our study-design. We decided to adopt the new inclusion criteria you suggested. In order to answer all the comments you gave us, we had changed the method, result, and discussion part as well as figures and tables.

*“I still find that representation of the data as it is presented in the manuscript is problematic although The authors have striven to accommodate my concerns. There were more cyst drainage and HAEs in the Tolvaptan group than the control group. The manuscript body should focus on the tolvapan and control group differences excluding the patents who had other volume reductive interventions, specifically cyst drainage and hepatic artery embolization so as that it represents impact of Tolvaptan on PKD and not the multiple volume reducing procedures.*

*The manuscript body should report the study findings after the exclusion of these cases with cyst drainage and HAE from both groups as they complicated/confound the analysis. The main result is shown in figure S3 representing the correct group of patients with PLD who did not have any other volume -reducing procedures. This analysis should be moved to the main manuscript as the main result. The current figure representing changes in TLV including those with other volume reducing procedures should be moved to supplemental data. Text in abstract, results section and discussion of the paper should be edited accordingly. Figure 1 flow diagram should be modified to should reflect that these cases with volumes reductive procedures were removed from both tolvaptan and control group analyses.”*

RESPONSE:

As you suggested, the inclusion criteria were modified to exclude the physical interventions for PLD and we have changed the method section, the flow diagram of figure1 according to the new inclusion criteria. In addition, we have changed the main result of figure 2 and 3 by using the sub-group analysis of the manuscript of first revision by using figure S3 and S4.

*“If all cyst drainage procedures are aggregated as opposed to being divided by indication (cyst infection versus routing drainage), one would expect there will be a difference between cases and controls. Please also summate these cases in tables 1 and 2 to determine whether they have statistical significance between the two groups. How much fluid was removed by these drainage procedures? Please add this volumes to tables 1 and 2.”*

RESPONSE: After we have collected the data of cyst drainage, we found that there was no statistical difference of the volume of cyst drainage between tolvaptan group and the non-tolvaptan group. These data were added in table S1 and figure S2.

*“The 16 with volume reducing procedures should be removed from the figure 4 the responder/ non responder main analysis in the main body of the paper.”*

RESPONSE: We have excluded the 16 patients from figure 4.

*“How was post menopausal defined? Please describe this in the methods.”*

RESPONSE: The menopausal status was determined by self-reported questionnaire. We added the method on page 8 line 8.

*“Page 14, line 13: Analysis of whole patients- change this as it is not good English”*

RESPONSE: We have changed the expression as following

“Analysis of the patients including the history of physical intervention for the polycystic liver

”

*“Supplemental figures have no labels as uploaded.”*

RESPONSE: We had added the labels of supplemental figures.

*“Figure S4- is this the correct title?”*

RESPONSE: We have corrected the titles of figure S3 and S4.

*******************************************************************************************

Again, thank you for giving us the valuable comments in order to strengthen our manuscript. We hope that these revisions persuade you to consider the acceptance of our submission.

Sincerely,

Hiroki Mizuno.

Nephrology Center

Toranomon Hospital Kajigaya, Japan

[hilomiz@yahoo.co.jp](mailto:hilomiz@yahoo.co.jp)

+08-44-3588-5111
